# Pharmacological targeting PTK6 inhibits the JAK2/STAT3 sustained stemness and reverses chemoresistance of colorectal cancer

**DOI:** 10.1186/s13046-021-02059-6

**Published:** 2021-09-22

**Authors:** Chaoqun Liu, Zhihua Pan, Qian Chen, Zetao Chen, Weiwei Liu, Ling Wu, Muhong Jiang, Wandie Lin, Yujie Zhang, Weihao Lin, Rui Zhou, Liang Zhao

**Affiliations:** 1 Department of Pathology, Nanfang Hospital, Southern Medical University, Guangzhou, China; 2 Department of Pathology, Guangdong Province Key Laboratory of Molecular Tumor Pathology, School of Basic Medical Sciences, Southern Medical University, Guangzhou, China

**Keywords:** Colorectal cancer, PTK6, Chemoresistance, Stemness, Small molecule kinase inhibitor

## Abstract

**Background:**

Chemoresistance is the major cause of chemotherapy failure in patients with colorectal cancer (CRC). Protein tyrosine kinase 6 (PTK6) is aberrantly overexpressed in clinical CRC tissues undergoing chemotherapy. We studied if PTK6 contributed to the chemoresistance of CRC in human and mice.

**Methods:**

We obtained tissue samples from patients with CRC and measured the expression of PTK6 by immunohistochemistry. Gain- and loss-of-function assays were performed to study the biological functions of PTK6. We constructed the FLAG-tagged wild type (WT), kinase-dead, and inhibition-defective recombinant mutants of PTK6 to study the effect phosphorylated activation of PTK6 played on CRC cell stemness and chemoresistance. We used small molecule inhibitor XMU-MP-2 to test the influence of PTK6 on sensitivity of CRC cells to 5-FU/L-OHP in both nude mouse and patient-derived xenograft (PDX) animal models.

**Results:**

PTK6 is overexpressed in CRC tissues and plays a stimulatory role in the proliferation and chemoresistance of CRC cells both *in vitro* and *in vivo*. PTK6, especially the phosphorylated PTK6, can promote the stemness of CRC cells through interacting with JAK2 and phosphorylating it to activate the JAK2/STAT3 signaling. Pharmacological inhibition of PTK6 using XMU-MP-2 effectively reduces the stemness property of CRC cells and improves its chemosensitivity to 5-FU/L-OHP in both nude mice subcutaneously implanted tumor model and PDX model constructed with NOD-SCID mice.

**Conclusions:**

PTK6 interacts with JAK2 and phosphorylates it to activate JAK2/STAT3 signaling to promote the stemness and chemoresistance of CRC cells. Pharmacological inhibition of PTK6 by small molecule inhibitor dramatically enhances the sensitivity to chemotherapy in nude mice and PDX models.

**Supplementary Information:**

The online version contains supplementary material available at 10.1186/s13046-021-02059-6.

## Background

Colorectal cancer (CRC) is the third most common cancer and the second cause of cancer-related death worldwide. Every year, there are more than 1.93 million newly developed colon cancer patients, accounting for 9.7 % of all new cancers diagnosed globally [1]. At present, surgical resection and chemotherapy are two major treatments for CRC [2]. Chemotherapy can palliate symptoms and prolong life of advanced CRC patients, but it fails to dramatically improve the clinical outcomes of patients with recurrent or metastatic chemoresistant CRC [3–5]. Therefore, a better understanding of molecular mechanisms underlying chemoresistance is essential for us to develop effective therapeutic strategies against CRC.

Protein tyrosine kinase 6 (PTK6), also known as breast tumor kinase (BRK), is a non-receptor intracellular tyrosine kinase primitively cloned from a metastatic breast tumor patient [6, 7]. Similar to other Src family kinases, PTK6 is composed of a Src homology 3 (SH3) domain, a Src homology 2 (SH2) domain and a tyrosine kinase domain [8, 9]. Since tyrosine kinases could participate in the regulation of many different signaling pathways [10, 11],the dysfunction of PTK6 often lead to the development and progression of tumors [8].

The aberrant expression of PTK6 has been identified in a variety of epithelial tumors including breast [12, 13], colon [14], non-small cell lung [15], pancreatic [16], prostate [17], ovarian [18] and gastric [19] cancer. PTK6 promoted the proliferation, survival and metastasis of breast cancer [20], and also contributed to its resistance to targeted therapies [21]. Activation of PTK6 facilitated the metastasis and impeded the therapy of hepatocellular carcinoma through promoting epithelial-mesenchymal transition and stemness [22]. PTK6 could mediate the PTEN loss induced invasion of prostate cancer through its kinase activity [23]. However, the roles of PTK6 on CRC progression remain controversial. Although PTK6 was elucidated to be a tumor suppressor in normal epithelia [24, 25], it promoted the azoxymethane-induced colon tumorigenesis in mouse models via activating STAT3 [26].

In this study, we focus on the positive correlation between the expression of PTK6 and chemoresistance of CRC patients, and attempt to determine the role of PTK6 on chemoresistance. The trial of small molecule PTK6 inhibitor is conducted on the patient-derived xenograft (PDX) animal model to study its role on chemosensitivity. Our results will disclose the functional mechanism of PTK6 in CRC chemoresistance, and reveal the therapeutic potential of targeting PTK6 in the clinical CRC treatment.

## Materials and methods

### Clinical CRC samples and cell cultures

Fresh primary CRC specimens with paired noncancerous colorectal tissues were obtained from the Tumor Tissue Bank of Nanfang Hospital. In each case, a diagnosis of primary CRC had been made before the elective surgery was carried out in Nanfang Hospital between 2017 and 2020. All of the CRC tissues obtained were kept in liquid nitrogen for further use. The Ethics Committee of Southern Medical University approved the study and all aspects of the study comply with the Declaration of Helsinki.

The normal human colon epithelial cell line NCM460, and colorectal cancer cell lines including LS174T, RKO, CACO2, SW620, SW480, HCT15, and DLD1 were purchased from the Cell Bank of the Chinese Academy of Sciences and maintained as previously described [27]. All of the cell lines used in this study obtain certificates within 4 years that authenticated by performing short tandem repeat (STR) profiling, and experiments were performed in cells propagated less than 6 months after resuscitation. RPMI 1640 (Hyclone) supplemented with 10 % fetal bovine serum (FBS) (Gibco-BRL, Invitrogen) was used to culture those cells at 37 °C with a humidity of 5 % CO_2_. Inhibitors including JAK2 inhibitor Ruxolitinib (Selleck) and PTK6 inhibitor XMU-MP-2 (WuXi AppTec) were added to cultured CRC cells for 24 h. Moreover, the susceptibility of CRC cells to chemotherapy drugs including 5-FU (Selleck) and Oxaliplatin (L-OHP, MCE) were studied.

Two pSuper.puro shRNA vectors for PTK6 were constructed in our laboratory based on the following sequence: sh1-PTK6 5’-CATCCATGGTTAAGTCATA-3’ and sh2-PTK6 5’-TGAAGAAGCTGCGGCACAA-3’. FLAG-tagged wild type (WT) PTK6 overexpression vector (pEnter-PTK6) was purchased from WZ Biosciences Inc. Kinase-dead PTK6^K219M^ variant, which has a lysine-to-methionine substitution at K219 in the ATP-binding site that results in a kinase dead mutant [28], and inhibition-defective PTK6^Y447F^ variant, which has a tyrosine-to-phenylalanine substitution at Y447 that results in a constitutively kinase activation, were purchased from GENEWIZ. CRC cells were transfected with PTK6 knockdown and overexpressing vectors using Lipofectamine 3000 Reagents (Invitrogen, CA) based on the manufacturer’s protocol. Stable PTK6 overexpression cell lines were obtained by antibiotic screening after transfection.

### Cell viability and Colony formation assays

The proliferation of CRC cells *in vitro* was measured using Cell Counting Kit-8 (Dojindo). According to the manufacturer’s instructions, cells were seeded into 96-well plates at 2 × 10^3^ per well in a final volume of 100 µL and cultured at 37 °C to obtain viable cells.

CRC cells were seeded directly into 6-well plates (5 × 10^2^ cells/well) for the colony formation assay and cultured in the presence of 10 % FBS at 37 °C with 5 % CO_2_. After 2 weeks, removed the medium and washed the plates three times with phosphate-buffered saline (PBS). The cells were fixed with anhydrous ethanol for 30 min and then stained with hematoxylin for 20 min. The total number of the colonies was counted. Three independent experiments were performed for both cell viability and colony formation tests. All the data was presented as means ± standard deviation.

### RNA preparation, cDNA synthesis and qPCR

Total RNA was extracted from CRC cell lines using RNAiso-Plus (TAKARA), and then reverse transcribed into cDNA using qRT-PCR cDNA synthesis kit (TAKARA). The real-time quantitative PCR was performed on the Applied Biosystems 7500 Sequence Detection system with TransStart Tip Green qPCR SuperMix (+ Dye II) (TransGen Biotech), 5 ng cDNA and 10 pM each primer. The cycling conditions were: 95 °C 5 min for 1 cycle; 95 °C 5 s, 60 °C 30 s, and 72 °C 34 s for 40 cycles, followed by the melting curve stage. Quantitative PCR primers were shown in Table S1. The mRNA level of target genes were normalized to the geometric mean of the housekeeping gene GAPDH and calculated via the 2-ΔΔCT method.

### Tumor sphere formation assays

CRC cells were seeded into 6-well ultralow attachment plates at a final concentration of 2 × 10^3^ cells per well, and re-suspended in the stem cell-conditioned medium containing 1640 medium (Invitrogen), 2 % B-27 Supplement (Invitrogen), 20 ng/ml basic fibroblast growth factor (bFGF, PeproTech), 20 ng/ml epidermal growth factor (EGF, SinoBiological), 0.4 % BSA (Sigma-Aldrich), and 5 µg/ml insulin (Sigma-Aldrich), at 37 °C with 5 % CO2 and saturated humidity for 12–14 days. Culture suspensions were passaged every 7 days when spheroid diameters were at least 50 μm. The images were taken by photography, and the number of cell spheres was counted. All experiments were conducted at least three times with three repeats for each treatment, and the data are presented as the mean ± standard deviation.

The in vitro limiting dilution assay (LDA) was performed to investigate self-renewal capacity of CRC cells. Primary CRC stem cell (CSC) spheres were collected and dissociated into single cells, then seeded into 96-well plates at a density of 5, 10, 20, 50, 100 or 200 cells per well. The formation of tumor spheres in each well was examined after 7 days, and wells without tumor spheres were counted. The sphere formation efficiency were calculated using the Extreme Limiting Dilution Analysis (http://bioinf.wehi.edu.au/software/elda) [29].

### Western blot (WB), Immunohistochemistry (IHC) and Immunofluorescence (IF)

Total proteins were extracted using RIPA lysis buffer with protease inhibitor cocktail, and quantified by BCA protein assay kit (Pierce, KeyGEN BioTECH) before separating by SDS-PAGE gel and transferring onto the PVDF membrane (Millipore). The membrane was blocked using Tris buffer containing 0.1 % Tween-20 and 5 % nonfat milk at 4 °C. Mouse antibodies against PTK6 (sc-16,617) were purchased from Santa Cruz Biotechnology (Santa Cruz, CA); Rabbit antibodies against JAK2 (3230), phosphorylated JAK2 (Y1007/1008, 3771), phosphorylated STAT3 (Y705, 9145), GAPDH (5174), Mouse monoclonal antibodies STAT3 (9139) were obtained from Cell Signaling Technology (MA, USA); Rabbit antibodies against phosphorylated-PTK6 (Tyr447, ab138368) were obtained from AbClonal Technology (MA, USA); Rabbit antibodies against phosphorylated-PTK6 (Tyr342, 09-144) were obtained from Millipore (Billerica, MA); Rabbit antibodies against Nanog (14,295), Sox2 (11,064), OCT4 (11,263), CD133 (66,666), Epcam (21,050), Sall4 (4500), Abcg2(27,286) was obtained from Proteintech Technology (Chicago, USA); Ki67 (ZM-0166) was obtained from ZSGB Technology (Beijing, China).The signal was detected with SuperSignal West Pico chemiluminescent Substrate (Thermo Scientific Pierce, Rockford, IL, USA) and visualized with tanon-5200 system (Bio-Tanon, Shanghai, China).

IHC was performed to investigate the expression of proteins in human colorectal cancer tissues. The sections were incubated overnight using primary antibodies at 4 °C. In this study, these slides were reviewed by 2 or 3 blind-folded pathologists. The IHC staining was scored semi-quantitatively as 0 (no staining), 1 (weak staining, light yellow), 2 (moderate staining, yellowish brown), and 3 (strong staining, brown) based on the intensity of staining. The IHC score of 2 and 3 was considered as high expression (or overexpression), whereas < 2 was regarded as low expression. The discrepancies (< 5 %) were resolved by simultaneous reevaluation.

CRC cells were seeded on confocal disks and cultured in complete medium for 12 h. After fixing in 4 % formaldehyde for 30 min, those cells were permeated by 0.2 % Triton-100 for 15 min. Then, cells were incubated with the primary antibody overnight, alexa 488/594 conjugated secondary antibody for 1 h, and followed by DAPI contrast staining for 10 min. Then observed by the Olympus confocal fluorescence microscope (Fluoview FV1000).

### Flow cytometry analysis

CRC cells were incubated with accutase and pipetted repeatedly to disperse into single cells. After washing twice with cold PBS, cells were centrifuged at 500×g for 5 min and re-suspended in binding buffer. Annexin V-FITC/PI (KGA107, Keygen Biotech, Jiangsu, China), Propidiumiodide (KGA512, Keygen Biotech, Jiangsu, China) or CD133 antibody (12-1338-42, eBioscience, invitrogen), SOX2 antibody (50-9811-82, eBioscience, invitrogen) were added to the cell suspension and incubated for 30 min at 4 °C. Then, the cells were re-suspended in PBS without washing and analyzed on a FACS flow cytometer according to the manufacturer’s instructions. The results were analyzed by FlowJo software.

### Subcutaneous, Orthotopic xenograft implantation models and in vivo limiting dilution assay in nude mice

For tumourigenesis assays, CRC cells (2 × 10^6^ cells per mouse) were subcutaneously injected into the right dorsal flanks of female BALB/c athymic nude mice (4–6 weeks of age, 18–20 g), which were obtained from the Animal Center of Southern Medical University, Guangzhou, China). After 4 weeks, mice were sacrificed by cervical dislocation and the xenograft tumors were quickly harvested for histological study. The tumor volume was calculated according to the formula: Volume (mm^3^) = width^2^ (mm^2^) × length (mm)/2. Tumor volume was measured every 3 days.

Approximately, 1 × 10^6^ HCT116 cells were injected into the cecal mesentry of BALB/c athymic nude mice at 6 weeks of age. After the construction of colorectal orthotopic implantation model, time of death was recorded for each mouse (7 mice per group).

For in vivo limiting dilution assays, subcutaneous xenograft were constructed using Vector、PTK6^WT^, PTK6^K219M^, and PTK6^Y447F^ cells (RKO) that were implanted at the gradient of 5 × 10^4^, 5 × 10^5^ and 5 × 10^6^ cells per mouse (*n* = 7 per group). Tumor formation incidence was calculated using the Extreme Limiting Dilution Analysis (http://bioinf.wehi.edu.au/software/elda/). All mice were raised under specific pathogen-free conditions, and all experiments were performed in accordance with the institutional guidelines approved by the Use Committee for Animal Care.

### PDX tumors and drug sensitivity assay

Human colorectal adenocarcinoma samples were processed within 30 min after surgical resection. Tumors were placed in sterile RPMI 1640 and minced into 3 × 3 × 3mm^3^ blocks on ice mechanically by scissors and then sterile scalpel. Non-necrotic tumor fragments were carefully selected and rinsed with serum-free PBS before implantation. After anesthesia, a 5mm incision was made on the upper abdominal skin of male NOD-SCID mice aged 4–5 weeks, and a total of 2–3 tumor blocks were implanted. Tumor growth in the mice was monitored weekly for 12–16 weeks. When the size of the established primary tumor model (P0) reached 1500 mm^3^, the primary tumor was cut into 3 × 3 × 3mm^3^ fragments and subcutaneously transplanted into the flank of 4–6 week-old male NOD-SCID mice for expansion (P1). This process is repeated in the process of expansion (P2 and P3). When the tumor volume increased to 100–200 mm^3^, mice were randomly divided into 4 groups (4–6 mice per group): DMSO; L-OHP; XMU-MP-2; XMU-MP-2 + L-OHP. All operations are performed aseptically in the SPF facility.

### Bioinformatics and statistical analyses

Microarray datasets were downloaded from public TCGA、GTEx and GEO public database. Three independent experiments were performed for each treatment and all data was presented as means ± standard deviation. Data were analyzed using SPSS software for Mac OS version 24.0. Unpaired Student’s t-test (two-tailed) was used for the comparison between unpaired two-groups and one-way ANOVA was applied for

multi-group data comparison. Bivariate correlation analysis (Pearson’s r test) was used to examine the correlation of 2 variables in human specimens. Kaplan–Meier and log-rank analyses were used for survival analysis. Spearman’s correlation analysis was performed to analyze correlated expression levels of PTK6 and chemoresistance/stemness related genes. *P* < 0.05 was considered significant, ***P < 0.001, **P < 0.01, *P < 0.05, ^**#**^ indicates *P* > 0.05.

## Results

### Overexpression of PTK6 is associated with the malignant progression of CRC

**Fig. 1:**
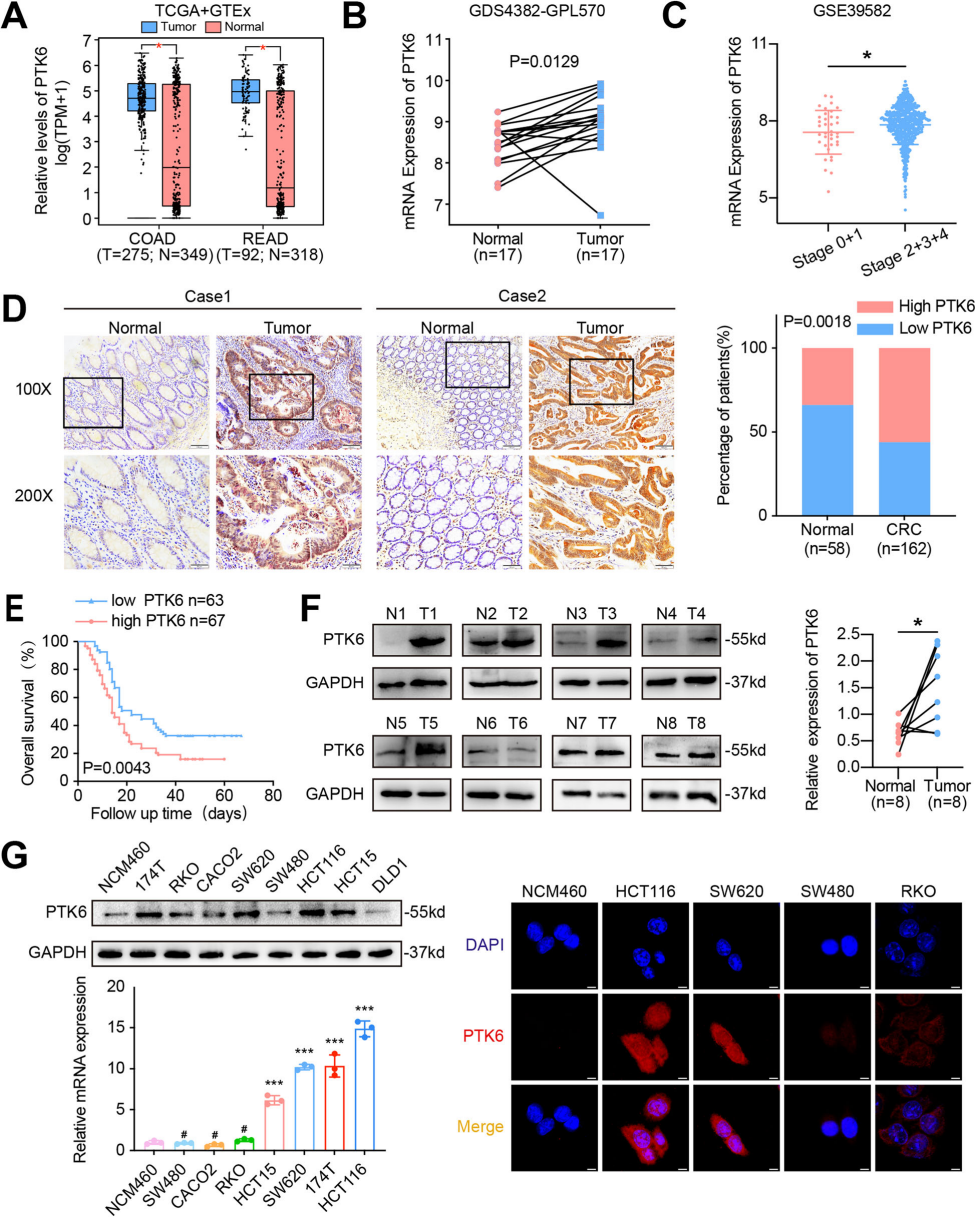
The overexpression of PTK6 is correlated with CRC progression. **A-B** The expression of PTK6 was analyzed in normal and CRC tissues from TCGA + GTEx and GEO (GDS4382-GPL570) databases. **C** The expression of PTK6 in stage I-IV CRC samples from GSE39582 dataset. **D** IHC analysis shows the expression of PTK6 in CRC tissues (T) and adjacent normal mucosa (N) of two representative cases. Bars in the right panel represent the percentage of high and low PTK6 expression patients. **E** The Kaplan-Meier survival curve shows the correatlation beteween PTK6 expression and overall survival (OS) in CRC patients from the Nanfang cohort. **F** Western blot analysis shows the expression of PTK6 in fresh CRC tissues (T) and adjacent non-tumor tissues (N). Scatter diagram on the right panel represents relative PTK6 expression in CRC tissues and paired normal tissues. **G** Western blot, real-time q-PCR and IF assays demonstrate the expression and localization of PTK6 in CRC cells. Scale bar represents 5 μm. *P < 0.05, ***P < 0.001,^**#**^ indicates *P* > 0.05

To investigate the role of PTK6 in CRC progression, we examined the expression of PTK6 in public microarray profile datasets including Gene Expression Omnibus (GEO), The Cancer Genome Atlas (TCGA) and Genotype-Tissue Expression (GTEx). The aberrantly elevated expression of PTK6 was detected in tumor tissues of CRC patients in both TCGA + GTEx and GEO (GDS4382-GPL570) databases (Fig. 1A and B). We also noted that PTK6 expression in stage II-IV CRC was significantly increased compared to stage 0-I CRC cases (Fig. 1C, GSE39582). Moreover, IHC results of clinical CRC samples collected from Nanfang hospital further demonstrated that high PTK6 expression was detected in 55.6 % (90/162) of the tumor tissues compared with that in 34.5 % (20/58) of the adjacent normal tissues (Fig. 1D). We also found a correlation between the high PTK6 expression and poor prognosis of CRC patients from Nanfang Cohorts (Fig. 1E). Consistent with the IHC results, western blot analysis also demonstrated a higher PTK6 expression in five out of eight fresh CRC tissues than the matched non-tumor tissues (Fig. 1F). We studied the expression of PTK6 in normal colon cells and seven different CRC cell lines. The expression of PTK6 was higher in 174T, HCT15, HCT116 and SW620 CRC cells at both transcriptional and translational levels than that in normal human colon epithelial NCM460 cells (Fig. 1G, left panel). Immunofluorescence staining showed that PTK6 was localized in both the cytoplasm and nucleus of CRC cells (Fig. 1G, right panel).

### PTK6 promotes the proliferation and chemoresistance of CRC cells

**Fig. 2:**
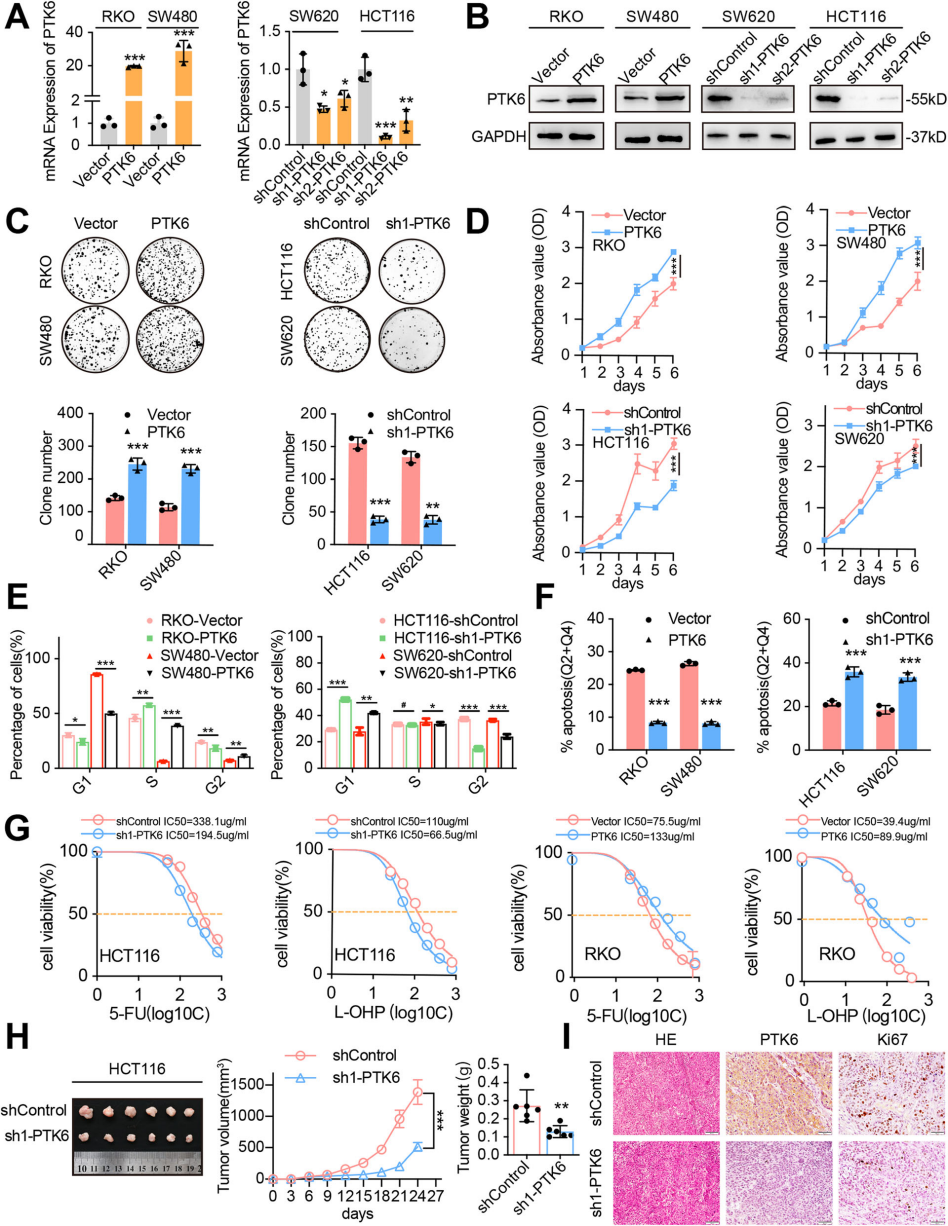
PTK6 promotes the proliferation and chemoresistance of CRC cells. **A-B** Real-time q-PCR and Western blot assays were performed to verify the successful construction of PTK6 overexpression and knockdown CRC cells. **C** Colony formation assays were performed to determine the effects of PTK6 on the growth of CRC cells. The number of colonies (> 50 cells) was scored (mean ± SD, *n* = 3). **D** CCK-8 assays were performed to determine the effects of PTK6 on the proliferation of CRC cells (mean ± SD, n = 3). (E) Flow cytometry analyses demonstrate the effect of PTK6 on the cell cycle progression (mean ± SD, *n* = 3). **F** Flow cytometry analyses show the effects of PTK6 on CRC cell apoptosis after treatment of 5-FU (100 µM) for 12 h(mean ± SD, *n* = 3). **G** Cell viability analyses were performed in PTK6 overexpression or silencing CRC cells after 5-FU and L-OHP treatment (mean ± SD, *n* = 3). **H** The subcutaneous tumors formed by control and PTK6 silencing HCT116 cells were obtained from nude mice. Both the volume and weight of subcutaneous tumors were shown in the right panel(mean ± SD, *n* = 3). (I) H&E and IHC staining were used to detect the expression of PTK6 and Ki67 in indicated subcutaneous tumors of nude mice. Scale bar represents 50 μm. *P < 0.05, **P < 0.01, ***P < 0.001

To investigate the biological effects of PTK6 on CRC cells, we transfected pEnter-PTK6 into RKO and SW480 cells, and successfully established two PTK6-overexpressing CRC cell lines (Fig. 2A-B, left panel). We also knocked down the endogenous expression of PTK6 in HCT116 and SW620 cells by introducing two short hairpin RNAs, which were named as sh1-PTK6 and sh2-PTK6. Sh1-PTK6 was chosen to carry out subsequent experiments for its higher interference efficiency compared with that of sh2-PTK6 in CRC cells (Fig. 2A-B, right panel). Immunofluorescence staining confirmed the successful construction of the PTK6 overexpressing or silenced CRC cells (Fig. S1A). Both CCK-8 and colony formation assays showed that overexpression of PTK6 significantly increased the growth and colony-forming ability of RKO and SW480 cells (Fig. 2C-D). Flow cytometry assays of both RKO and SW480 cells with PI staining indicated that PTK6 overexpression increased the proportion of S phase cells, decreased the proportion of G1/G0 phase cells, and reduced the extent of apoptosis (Fig. 2E-F and Fig. S1B-C). In contrast, knockdown of PTK6 significantly decreased the proliferation, colony formation, and cell-cycle progression capacities of CRC cells, while increasing the extent of apoptosis (Fig. 2 C-F and Fig. S1B-C).

Overexpression of PTK6 increased the viability of 5-FU/L-OHP treated RKO and SW480 cells, while knockdown of PTK6 inhibited the viability of 5-FU/L-OHP treated HCT116 and SW620 cells (Fig. 2G and Fig. S1D). The volume and weight of the subcutaneous tumors formed by control cells were at least two times bigger than those formed by PTK6 knockdown HCT116 cells on the day of dissection (Fig. 2H). IHC staining with Ki-67 confirmed a lower proliferation index in subcutaneous tumors formed by PTK6 silenced HCT116 cells than in those formed by the control cells (Fig. 2I). These data indicate that PTK6 contributes to the proliferation and chemoresistance of CRC cells.

### Knockdown of PTK6 inhibits the stemness of CRC cells

**Fig. 3:**
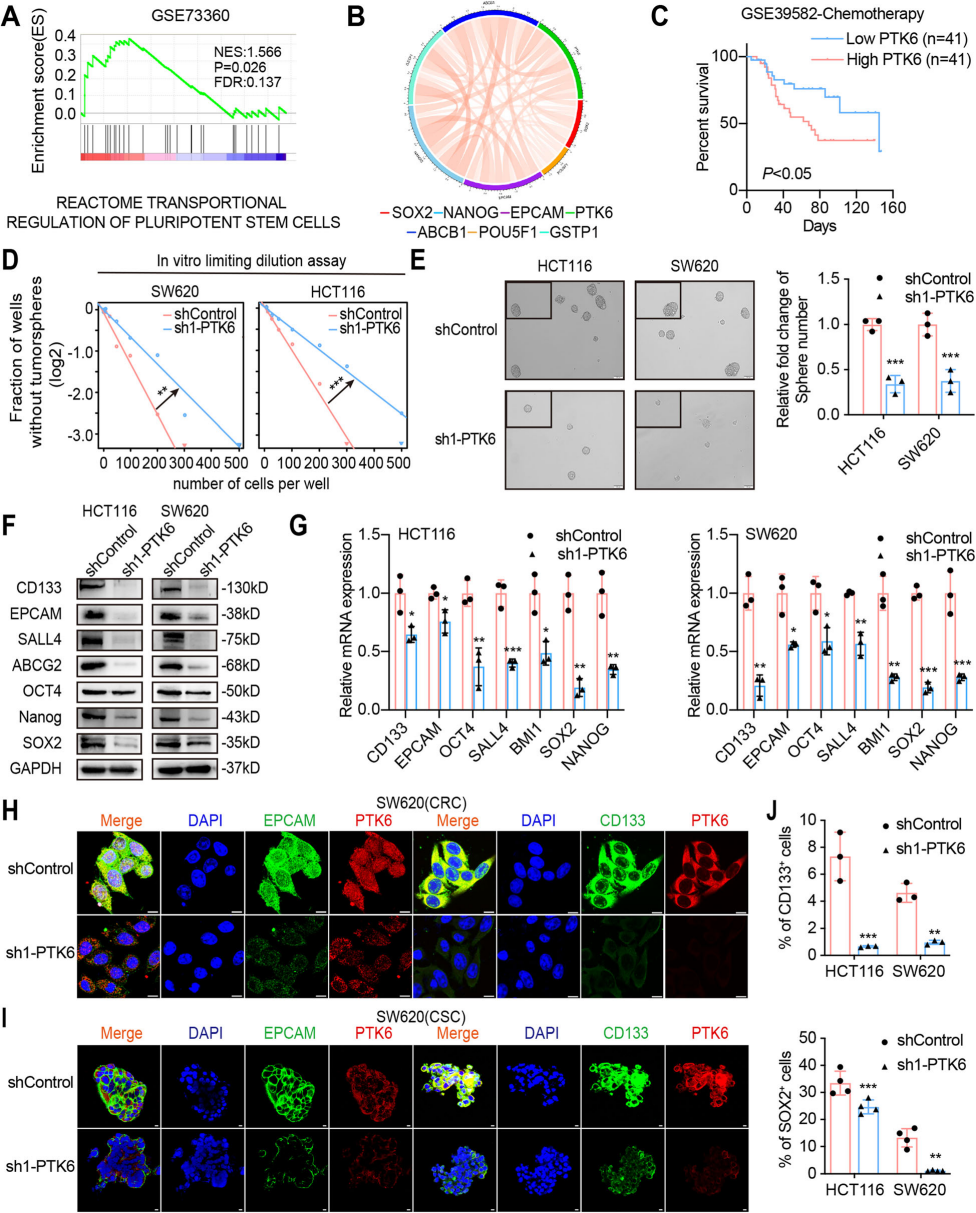
Knockdown of PTK6 inhibits the stemness of CRC cells. **A** The GSEA result indicates an enrichment of gene sets related to reactome transportional regulation of pluripotent stem cells in PTK6 overexpression CRC tissues. **B** The correlations of PTK6 with chemoresistance and stemness related genes were detected from the GEO database. **C** Kaplan–Meier survival curves in CRC patients undergoing chemotherapy with distinct PTK6 expression levels. **D** The in vitro limiting dilution assay shows the effects of PTK6 on the formation of CSC spheres (mean ± SD, *n* = 12), likelihood ratio test. **E** Tumor sphere formation assays indicate that silencing of PTK6 decreases the sphere formation of CSC cells. Scale bar represents 50 μm. The right panel shows the relative fold change of sphere number (mean ± SD, *n* = 3). **F** Western blots assays demonstrate the influence of PTK6 on the expression of stem cell markers in CRC cells. **G** Real-time q-PCR analysis was used to detect the effect of PTK6 on the expression of stem cell markers and chemoresistance related genes in CRC cells (mean ± SD, *n* = 3). **H** Co-immunofluorescent staining of PTK6 (red), CD133 (green) and EPCAM (green) in CRC cells. Scale bar represents 5 μm. **I** Co-immunofluorescent staining of PTK6 (red), CD133 (green) and EPACAM (green) in tumor spheres. Scale bar represents 5 μm. **J** The number of CD133^+^ or SOX2^+^ cells was evaluated in the control and PTK6 silencing CRC cells by flow cytometry (mean ± SD, *n* = 3). *P < 0.05, **P < 0.01, ***P < 0.001

To explore the mechanisms underlying PTK6 regulated chemoresistance in CRC, we applied gene set enrichment analysis (GSEA) on the microarray data from GSE73360. The GSEA results indicated that stemness regulation (Fig. 3A) and chemotherapy tolerance related pathways (Fig. S2A) were enriched in the PTK6-overexpresssing CRC tissues. In addition, we found that the PTK6 expression was positively correlated with chemoresistance related genes (GSTP1 and ABCB1) and stemness related genes (NANOG, EPCAM, POU5F1, and SOX2) in dataset GSE103479 (Fig. 3B). Consistent with the result from GEO dataset, we also detected a positive correlation between PTK6 expression and stemness related genes including ABCG2, BMI1, EPACM, POU5F1, and SALL4 after analyzing CRC data from TCGA (Fig. S2B). Moreover, the Kaplan-Meier analysis of dataset GSE39582 showed that PTK6 overexpression was correlated with the poor overall survival (OS) of CRC patients receiving chemotherapy (Fig. 3C). These results suggested that the aberrant expression of PTK6 was closely related to CRC stemness and chemoresistance. The *in vitro* limiting dilution assays were conducted to investigate the role of PTK6 in maintaining the stemness property. Knockdown of PTK6 significantly decreased the self-renewal capacity of HCT116 and SW620 cells (Fig. 3D and Fig. S2C). In addition, fewer and smaller CSC spheres were formed in the PTK6 knockdown HCT116 and SW620 cells (Fig. 3E and Fig. S2D). The qPCR, western blotting, and IF assays demonstrated that knockdown of PTK6 could decrease the expression of stemness markers (CD133, EPCAM, OCT4, SALL4, SOX2, and NANOG), and drug resistance factors (ABCB1, GSTP1, TOP2A, ABCC1 and ABCG2) in both HCT116 and SW620 cells (Fig. 3F-G, H and Fig.S2E). We also detected an obvious co-localization of PTK6 and stemness markers in CSC spheres, and knockdown of PTK6 decreased the expression of CD133 and EPCAM in CSC spheres formed by SW620 cells (Fig. 3I). Besides, flow cytometry assays showed that the number of CD133^+^and SOX2^+^ cells was decreased in the PTK6 silencing HCT116 and SW620 cells (Fig. 3J and Fig. S2F-G). In conclusion, we observed that PTK6 contributed to the stemness of CRC cells.

### The phosphorylation of PTK6 enhances CRC cell stemness

**Fig. 4:**
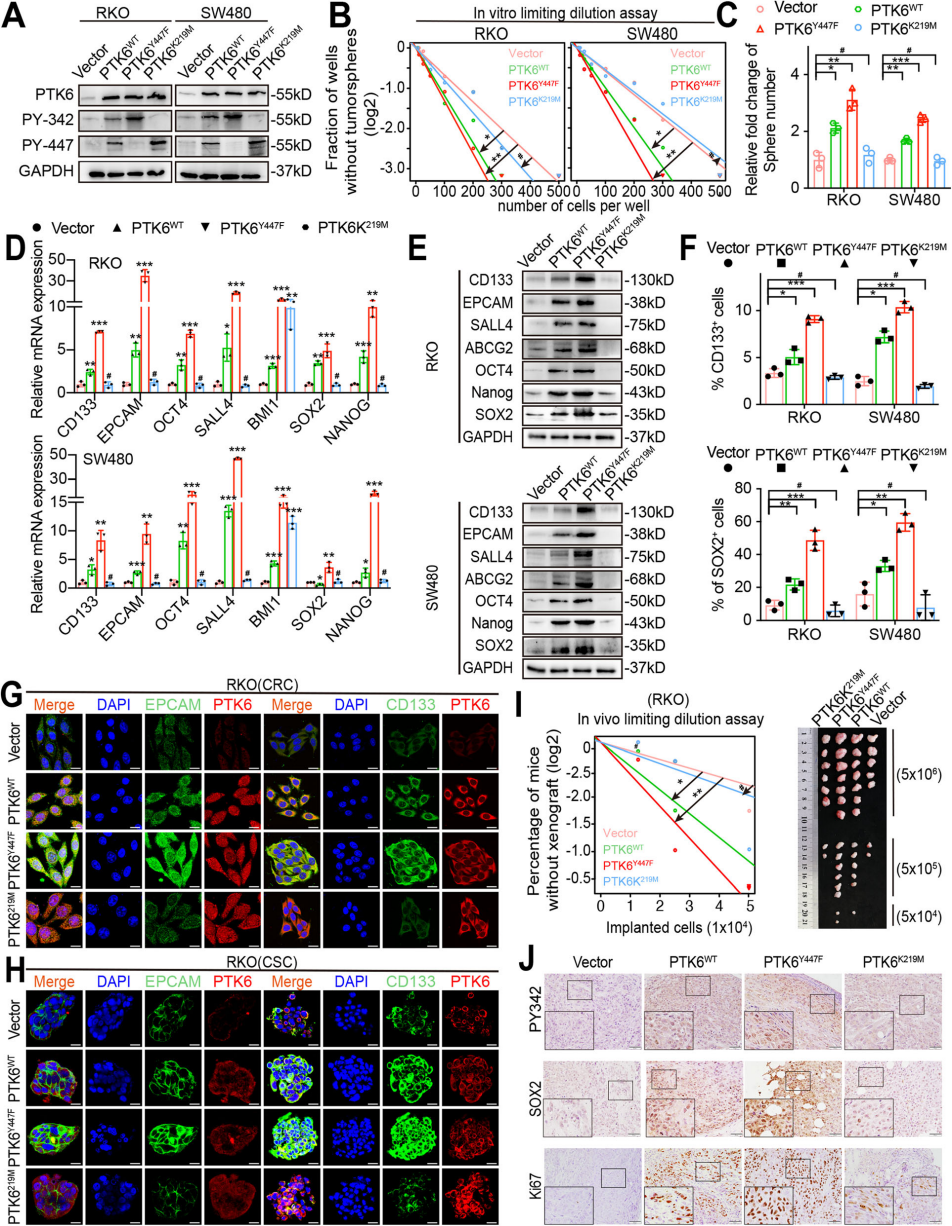
The phosphorylation of PTK6 promotes CRC cell stemness. **A** Western blot analyses were performed to verify the successful construction of FLAG-tagged empty vector, wild-type (WT), kinase-dead (KM) and constitutively active (YF) PTK6 recombinant mutants overexpression CRC cells. **B** *In vitro* limiting dilution assays show the effects of phosphorylated PTK6 on the formation of CSC spheres (mean ± SD, *n* = 12), likelihood ratio test. **C** Tumor sphere formation assays were performed to detect the sphere-forming ability of vector, PTK6^WT^, PTK6^KM^ and PTK6^YF^ overexpression CRC cells (mean ± SD, *n* = 3). **D** Real-time q-PCR analysis shows the effects of phosphorylated PTK6 on the expression of stem cell markers  in CRC cells (mean ± SD, *n* = 3). **E** Western blot assays were performed to detect the effects of phosphorylated PTK6 on the expression of stem cell markers in CRC cells. **F** The number of CD133 + or SOX2 + cells was evaluated in the vector, PTK6^WT^, PTK6^KM^ and PTK6^YF^ overexpression CRC cells by flow cytometry (mean ± SD, *n* = 3). **G** Co-immunofluorescent staining of PTK6 (red), CD133 (green) and EPACAM (green) in CRC cells. Scale bar represents 5 μm. **H** Co-immunofluorescent staining of PTK6 (red), CD133 (green) and EPACAM (green) in CRC tumor spheres. Scale bar = 5 μm. **I** In vivo limiting dilution analysis was performed to detect the tumorigenicity of vector, PTK6^WT^, PTK6^KM^ and PTK6^YF^ overexpression CRC cells in nude mice (mean ± SD, *n* = 7). **J** IHC staining was used to detect the expression of PY342, SOX2 and Ki67 in indicated subcutaneous tumors of nude mice. Scale bar represents 50 μm. *P < 0.05, **P < 0.01, ***P < 0.001, ^**#**^ indicates *P* > 0.05

Since the phosphorylation of PTK6 at tyrosine 342 (PY342) could activate, while at tyrosine 447 (PY447) could inhibit the activity of PTK6, we constructed the FLAG-tagged wild type (PTK6^WT^), kinase-dead (PTK6^K219M^), and inhibition-defective (PTK6^Y447F^) recombinant PTK6 variants, and transiently transfected them into SW480 and RKO cells (Fig. 4A). Overexpression of PTK6^Y447F^ resulted in a constant phosphorylation at tyrosine residue 342 in SW480 and RKO cells. The *in vitro* limiting dilution and sphere formation assays showed that both PTK6^WT^ and PTK6^Y447F^ could promote the self-renewal of RKO and SW480 cells. However, PTK6^Y447F^ displayed a more prominent effect compared with PTK6^WT^, indicating that the phosphorylated activation of PTK6 was essential for the stemness maintenance (Fig. 4B-C and Fig. S3A). In contrast, the PTK6^K219M^ that constantly phosphorylated at tyrosine 447 showed no effect on the self-renewal of RKO and SW480 cells (Fig. 4B-C and Fig. S3A). Consistently, it was not PTK6^K219M^, but PTK6^WT^ and PTK6^Y447F^, that could increase the expression of the stemness markers and drug resistance factors (Fig. 4D-E and Fig. S3B). Flow cytometry assays showed that the number of CD133^+^,SOX2^+^ cells was dramatically increased in the PTK6^Y447F^ transfected RKO and SW480 cells compared with PTK6^WT^ and PTK6^K219M^ overexpression cells (Fig. 4F and Fig. S3C). We also found that the expression of CD133 and EPCAM was dramatically increased in the PTK6^Y447F^ transfected CSC spheres after IF staining (Fig. 4G-H). The limiting dilution xenografted assays indicated that both the PTK6^WT^ and PTK6^Y447F^ could increase the tumorigenic capacity of RKO cells *in vivo*, but the performance of PTK6^Y447F^ wass much better than that of PTK6^WT^ (Fig. 4I). Additionally, IHC results demonstrated a higher SOX2 and Ki67 expression level in subcutaneous tumors formed by the PTK6^Y447F^ overexpression RKO cells, compared with those formed by the PTK6^WT^ and PTK6^K219M^ overexpression cells (Fig. 4 J). To sum up, the phosphorylated activation of PTK6 was required for the PTK6 promoted CRC cell stemness.

### PTK6 promotes CRC progression by activating JAK2/STAT3 signaling

**Fig. 5:**
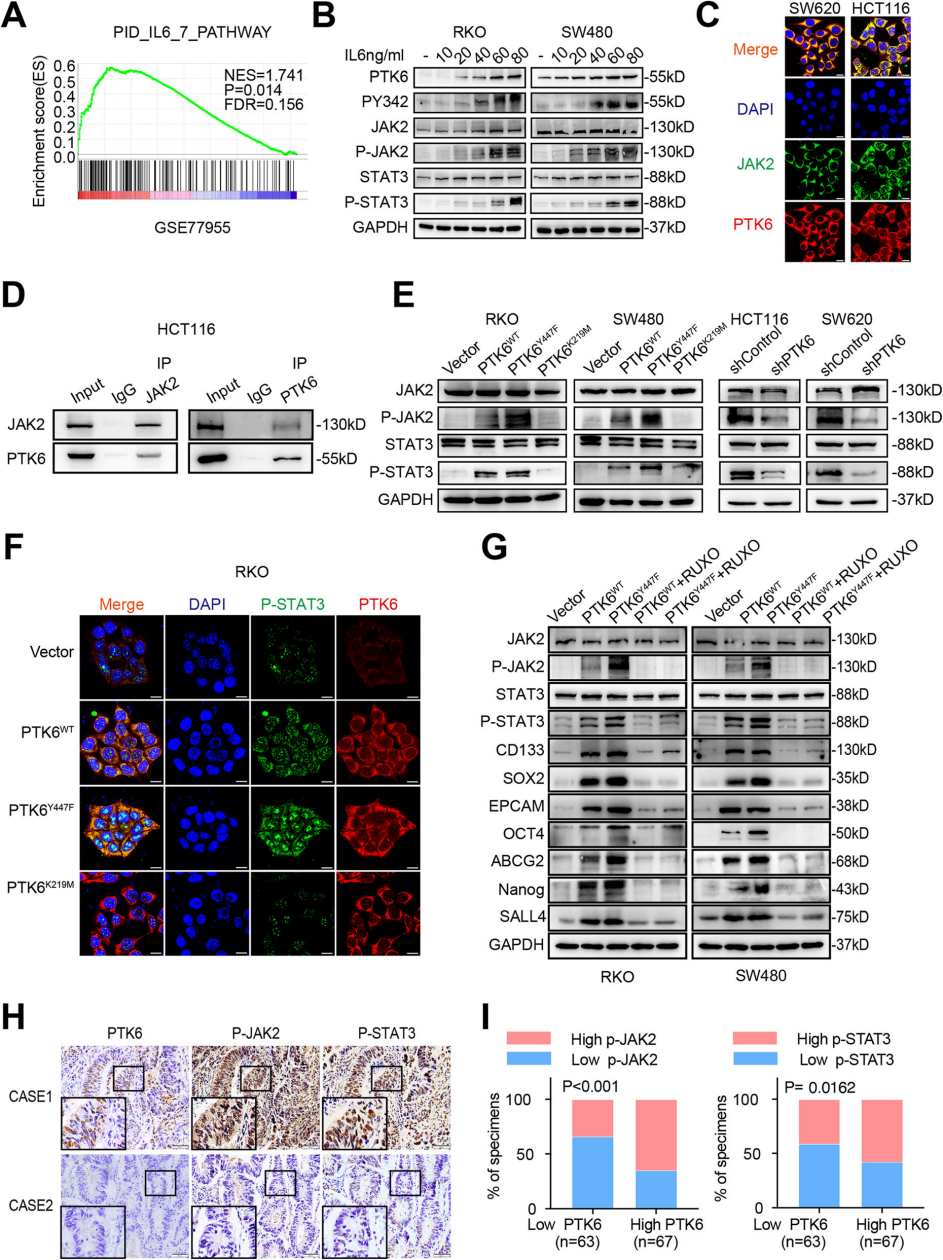
PTK6 promotes CRC progression by activating JAK2/STAT3 signaling pathway. **A** The GSEA result indicates an enrichment of gene sets related to IL6 signaling pathway in PTK6 overexpression group. **B** Western blot assays indicate that IL6 can stimulate the activation of the PTK6-JAK2/STAT3 pathway in a dose-dependent manner in CRC cells. **C** The co-localization of JAK2 (green) and PTK6 (red) in CRC cells was assessed by IF staining. The scale bar represents 5 μm. **D** Co-IP results show an endogenous protein interaction between PTK6 and JAK2 in CRC cells. **E** Western blot assays show that the phosphorylation of PTK6 plays a vital role in the activation of the JAK2/STAT3 pathway. **F** The co-localization of p-STAT3 (green) and PTK6 (red) in vector, PTK6^WT^, PTK6^KM^ and PTK6^YF^ overexpression CRC cells were assessed by IF staining. The scale bar represents 5 μm. **G** Western blot analyses show the expression of JAK2/STAT3 pathway members and stem cell markers in vector, PTK6^WT^, PTK6^KM^ and PTK6^YF^ overexpression CRC cells with or without the treatment of RUXO. **H** Representative immunohistochemical (IHC) staining images of CRC tissues from the Nanfang cohort show the expression of PTK6, p-JAK2 and p-STAT3 in CRC and the adjacent normal tissues. Scale bars represent 50 μm. **I** Histograms show that the expression of PTK6 is correlated with that of p-JAK2 and p-STAT3 in CRC tissues

We performed GSEA on microarray data GSE77955 and found the enrichment of IL6 signaling in PTK6-overexpressed CRC tissues (Fig. 5A). Consistent with the GSEA result, recombinant IL6 could not only stimulate the expression of PTK6, but also activate PTK6 through phosphorylation at site Y342 in a dose dependent manner. Meanwhile, we also noted the activation of the JAK/STAT3 signaling, which was a well-known downstream of IL6 (Fig. 5B). IF assays showed the co-localization of PTK6 and JAK2 in HCT116 and SW620 cells (Fig. 5C). The following co-immunoprecipitation analyses further demonstrated a physical interaction between PTK6 and JAK2 (Fig. 5D).

In PTK6^WT^ and PTK6^Y447F^ transfected RKO and SW480 cells, we detected an increased phosphorylation of JAK2 and STAT3. In contrast, knockdown of PTK6 suppressed the phosphorylation of JAK2 and STAT3 in HCT116 and SW620 cells (Fig. 5E). Besides, IF staining also indicated an increased intracellular STAT3 phosphorylation in PTK6^WT^ and PTK6^Y447F^ transfected RKO and SW480 cells (Fig. 5F). Ruxolitinib, an inhibitor of the JAK1/2, could attenuate the PTK6 stimulated phosphorylation of JAK2 and STAT3, as well as PTK6 increased expression of stemness-related genes in RKO and SW480 (Fig. 5G). In addition, we found a positive correlation between the expression of STAT3 and stemness-related genes, including ABCG2, CD133, EPCAM, OCT4, SALL4, SOX2 and NANOG in CRC specimens from the GSE39582 and GSE17538 cohorts (Fig.S4A). IHC assays showed that PTK6 was also positively correlated with the phosphorylation of JAK2 and STAT3 in CRC samples from Nanfang cohort (Fig. 5H-I). Kaplan-Meier survival analysis showed that CRC patients with high p-JAK2 and p-STAT3 levels had worse prognosis than those with low p-JAK2 or p-STAT3 levels (Fig. S4B). In summary, we found that PTK6 could interact with JAK2 and phosphorylate it to promote the stemness of CRC cells.

### XMU-MP-2 inhibits the PTK6 induced proliferation, stemness, and chemoresistance of CRC cells

**Fig. 6:**
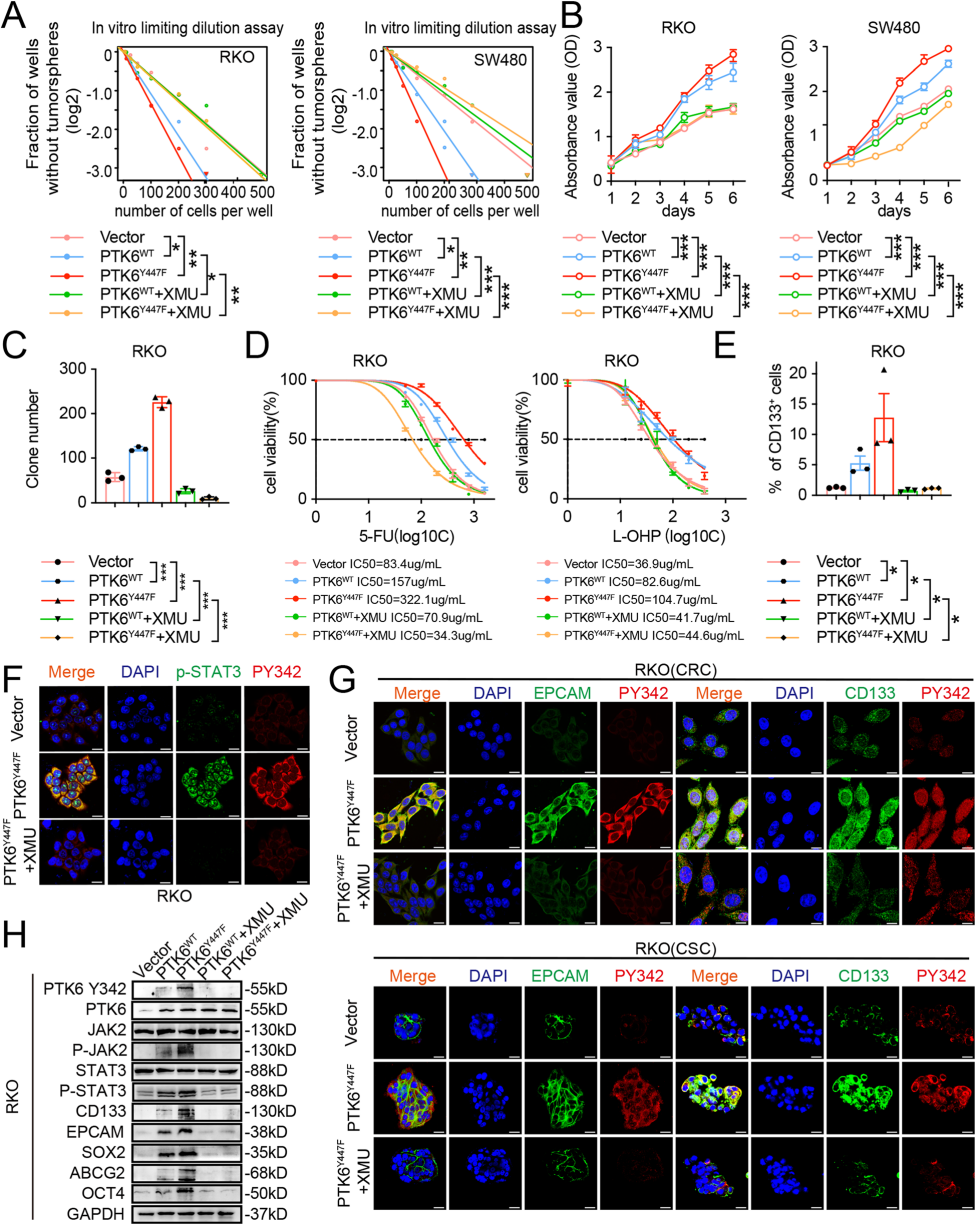
XMU-MP-2 inhibits the PTK6 induced proliferation, stemness, and chemoresistance of CRC cells. **A** The *in vitro* limiting dilution assays show the effects of XMU-MP-2 on the PTK6 enhanced formation of tumor spheres (mean ± SD, *n* = 12), likelihood ratio test. **B** CCK-8 assays were performed to determine the effects of XMU-MP-2 on the PTK6 induced proliferation CRC cells (mean ± SD, *n* = 3). **C** Colony formation assays were performed to determine the effects of XMU-MP-2 on PTK6 induced growth of CRC cells. The number of colonies (> 50 cells) was scored (mean ± SD, *n* = 3). **D** Dose–response curves show the effects of XMU-MP-2 on the sensitivity of vector, PTK6^WT ^and PTK6^YF^ overexpression CRC cells to chemotherapy drugs (mean ± SD, *n* = 3). **E** The numbers of CD133 + cells in indicated CRC cells were evaluated by flow cytometry (mean ± SD, *n* = 3). **F** Immunofluorescence staining of CRC cells shows that XMU-MP-2 can inhibit the phosphorylation of PTK6, as well as the activation of its downstream STAT3 signaling. **G** Immunofluorescence staining of CRC cells and CSC spheres shows that XMU-MP-2 can inhibit the phosphorylation of PTK6 and the expression of stem cell markers. **H** Western blot assays indicate that XMU-MP-2 can inhibit the PTK6 induced activation of JAK2/STAT3 signaling and the expression of stem cell markers. *P < 0.05, **P < 0.01, ***P < 0.001

XMU-MP-2 is a small-molecule PTK6 inhibitor that could selectively inhibit the kinase activity of PTK6 [30]. In PTK6-transfected RKO and SW480 cells, we found that XMU-MP-2 could inhibited the auto-phosphorylation of PTK6 at Y342, doing so in a dose-dependent manner with maximum inhibition observed at 5000 nmol/L (Fig. S5A). Our results showed that XMU-MP-2 could block the proliferation and viability of RKO and SW480 cells, with IC50 values at 447.5nmol/L and 293.8 nmol/L, respectively (Fig. S5B). XMU-MP-2 obviously decreased the PTK6-induced stemness, proliferation, and chemoresistance of CRC cells (Fig. 6 A-E and Fig. S5C-D). Furthermore, administration of XMU-MP-2 totally abolished the PTK6 variants stimulated phosphorylation of JAK2 and STAT3. XMU-MP-2 could also attenuate the PTK6 variants stimulated expression of stemness-related genes (Fig. 6 F-H). Altogether, these results indicated that XMU-MP-2 could suppress CRC stemness and enhance sensitivity through targeting PTK6.

### XMU-MP-2 enhances the chemosensitivity of CRC cells *in vivo*

**Fig. 7:**
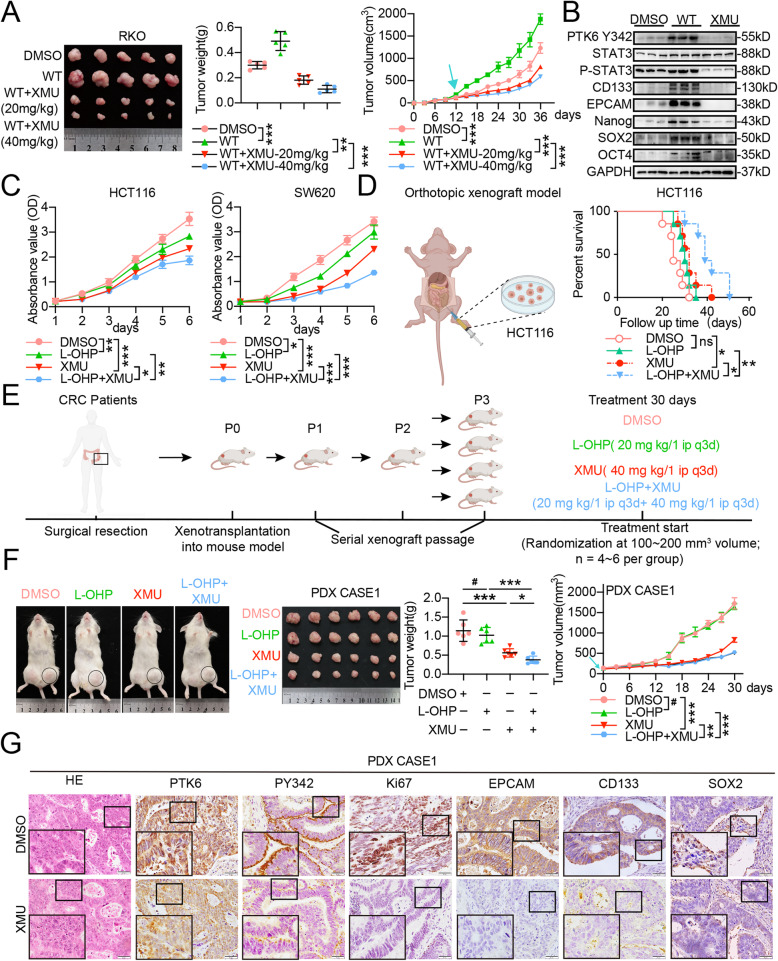
XMU-MP-2 promotes the chemosensitivity of CRC *in vivo*. **A** XMU-MP-2 suppresses the weight and volume of the subcutaneous tumors formed by PTK6 overexpression CRC cells. Both the weight and volume of subcutaneous tumor were shown in the right panel (mean ± SD, *n* = 5). **B** Western blot assays indicate that XMU-MP-2 can inhibit the PTK6 induced activation of STAT3 signaling and the expression of stem cell markers in the subcutaneous tumors. **C** CCK8 assays show that XMU-MP-2 can further inhibit L-OHP suppressed proliferation of CRC cells (mean ± SD, *n* = 3). **D** The orthotopic xenograft tumor model reveals that XMU-MPX-2 can prolong the overall survival of the L-OHP treated tumor-bearing mice (mean ± SD, *n* = 7). **E** The construction process of patient-derived xenograft (PDX) tumors. **F** XMU-MP-2 suppresses the weight and volume of the PDX tumors derived from CASE1 in the L-OHP treated tumor-bearing mice. Both the volume and weight of subcutaneous tumor were shown in the right panel (mean ± SD, *n* = 6). **G** IHC staining indicates that XMU-MP-2 can reduce the phosphorylation of PTK6 and the stem cell markers in the PDX tumors derived from CASE1. *P < 0.05, **P < 0.01, ***P < 0.001, ^**#**^ indicates *P* > 0.05

Next, we examined the effects of XMU-MP-2 on the chemosensitivity of CRC in  vitro. We introduced XMU-MP-2 to subcutaneous xenograft model of nude mice, and found that XMU-MP-2 dramatically suppressed the weight and volume of subcutaneous tumors formed by PTK6^WT^ transfected CRC cells (Fig. 7A). Western blot analyses showed that XMU-MP-2 neutralized the exogenous PTK6 stimulated phosphorylation of PTK6 and STAT3, as well as the expression of stemness-related genes in subcutaneous tumors (Fig. 7B). IHC staining showed that phosphorylated activation of PTK6 in subcutaneous tumors and clinical CRC tissues was accompanied with the activation of STAT3 signaling and the increased expression of stemness-related genes. (Fig. S6A-B). Then, we tested the efficacy of XMU-MP-2 monotherapy, L-OHP monotherapy, the XMU-MP-2 and L-OHP combined therapy on the growth of subcutaneous tumors. Cell viability assays showed that the combined therapy further suppressed the proliferation of HCT116 and SW620 cells inhibited by either XMU-MP-2 or L-OHP monotherapy (Fig. 7C). Consistent with the *in vitro* experiments, combined therapy of XMU-MP-2 and L-OHP significantly reduced the volume, weight and proliferation index of CRC subcutaneous xenograft tumors compared with the XMU-MP-2 or L-OHP monotherapy (Figure S6C-D). The survival analysis revealed that it was XMU-MP-2 monotherapy, rather than L-OHP monotherapy, that could lead to a longer overall survival of orthotopic transplanted nude mice. Moreover, the combined therapy also displayed a prolonged overall survival compared to those treated by either XMU-MP-2 or L-OHP monotherapy (Fig. 7D). Patient-derived xenografts (PDX) are similar to primary tumors and commonly used to study the therapeutic responses of transformed anticancer drugs [31]. In this study, we successfully established the PDX model using tumor tissues derived from two CRC patients (Fig. 7E). The combined XMU-MP-2 and L-OHP therapy could effectively reduce the volume and weight of the PDX tumors (Fig. 7F and Fig. S6E). The expression and phosphorylation of PTK6, the expression of stemness markers, and the Ki67 index were suppressed in the XMU-MP-2 treated PDX tumors (Fig. 7G and Fig. S6F). Taken together, these results indicated that XMU-MP-2 improved the sensitivity of chemotherapy in both nude mice and PDX models.

## Discussion

Chemotherapy is a primary clinical treatment for CRC, but chemoresistance dramatically impedes the efficacy of chemotherapy. In this research, we verified PTK6 as a chemoresistance-related gene in CRC for the first time. We found that PTK6 could interact with JAK2 and activate JAK2/STAT3 signaling to promote the stemness and chemoresistance of CRC. We also confirmed that kinase-dependent activity was required for the PTK6 mediated stemness and chemoresistance in CRC cells. Most importantly, we proposed an applicable prospect for small molecule PTK6 inhibitors in CRC clinical treatment.

The aberrant expression of PTK6 has been identified in multiple malignant tumors including breast, colon, head and neck, ovary, prostate, lung, bladder, bile duct, pancreas, and gastric cancer, as well as T- and B-cell lymphoma [7, 32]. However, the function of PTK6 in different kinds of tumors remains controversial. PTK6 promoted the proliferation, survival, and metastasis of breast cancer [20]. The expression of PTK6 was also associated with the poor prognosis of patients with non-small cell lung cancer and ovarian cancer [15, 18]. In contrast, PTK6 played an inhibitory role in the regulation of the proliferation, differentiation, and migration of nasopharyngeal carcinoma and esophageal squamous cell carcinoma [33, 34]. Although tissue specific roles of PTK6 have been discovered, no consistency has been reached in CRC. Priya S. Mathur et al. demonstrated a decreased PTK6 expression in the development of CRC [28]. However, we identified a stimulatory role of PTK6 on the proliferation, stemness and chemoresistance of CRC cells. Our finding is consistent with the study of Wei-Shone CHEN et al. that PTK6 expression was dramatically increased in human intestinal tumors [35].

In this manuscript, we noted that PTK6 was correlated with the poor prognosis of CRC patients undergoing chemotherapy, and PTK6 played a stimulatory role on the viability of 5-FU/L-OPH treated CRC cells. To explore the mechanisms underlying PTK6-mediated chemoresistance, we detected an enrichment of stemness-related pathways in PTK6 overexpression CRC tissues from open access database. It is consistent with the fact that stemness contributes to the tumorigenic and chemoresistant phenotype in many cancer types [36–38]. Moreover, a series of functional experiments also demonstrated that PTK6 could dramatically stimulate the expression of stemness-related genes and increase the formation of CSC spheres. As a result, PTK6 might enhance CRC chemoresistance through regulating stemness.

PTK6 is a ubiquitous non-receptor intracellular tyrosine predominately located in the cytoplasm [14]. As a member of Src family kinases, PTK6 is composed of a Src homology 3 (SH3) domain, a Src homology 2 (SH2) domain and a tyrosine kinase domain. Therefore, PTK6 probably performs its functions through synergetic partnerships with other tumorigenic factors mediated by SH2/SH3 domains, and/or phosphorylation accomplished by the tyrosine kinase domain. To confirm the hypothesis, we constructed two PTK6 variants, and found that only the PTK6 constant activated PTK6^Y447F^ variant could stimulate the expression of stemness markers and increase the resistance to chemotherapy, while PTK6 phosphorylation silenced PTK6^K219M^ variant could not influence them at all. Hence, PTK6 regulated CRC stemness and chemoresistance mainly through its kinase activity. In addition, wild type PTK6 and PTK6^Y447F^ variant could enhance the phosphorylation of JAK2, proliferation and stemness of CRC cells, while the PTK6^K219M^ variant did not have such effects. We also proved the interaction between PTK6 and JAK2. Thus, PTK6 could interact with JAK2 at SH2/SH3 domain and phosphorylated activate JAK2 to enhance CRC proliferation and stemness.

Many cytokines, such as epidermal growth factor (EGF) and insulin-like growth factor (IGF), stimulate the phosphorylated activation of PTK6 [39, 40]. In this study, IL6 signaling was enriched in the PTK6-overexpressing CRC tissues by GSEA. IL-6 is a pro-survival signaling molecule released by non-tumor cells harboring or surrounding the tumor microenvironment. The aberrant IL-6 expression contributes to the profound consequences in metastasis, resistance, and stemness properties of cancer cells [41–43]. The abnormal activation of the IL-6-JAK-STAT3 signaling in cancer cells has emerged as an important mechanism for the initiation, development, and progression of various tumor types [44]. In addition, IL6 could nourish the development of stemness properties through activating the JAK/STAT3 signaling [45]. STAT3 is a transcription factor that could activate the expression of several multi-drug resistant (MDR) genes and result in the chemoresistance of breast cancer cells [46]. Here, we also confirmed a stimulatory role of IL-6 on the phosphorylated activation of PTK6, which subsequently enhanced the stemness and chemoresistance of CRC through activating the JAK2/STAT3 signaling. Although it has been reported that PTK6 could phosphorylate STAT3 at Tyr705 [26], we verified that JAK2 was also a direct substrate of PTK6 in the current study.

Recently, several small-molecule inhibitors specifically target on PTK6 have been developed to inhibit its phosphorylation and downstream signaling [47]; XMU-MP-2, a ATP-site-directed kinase inhibitor with mild cytotoxicity, exhibited a strong inhibition on the growth of breast cancer through selectively inhibiting the activity of tyrosine kinases with SRC and FRK domain [30]. The combined treatment of XMU-MP-2 and HER2/ER inhibitor showed a synergistic effect on the proliferation of breast cancer. In our study, we found that targeting PTK6 using XMU-MP-2 remarkably attenuated the stemness and increased the chemotherapy sensitivity of CRC in both nude mice and PDX models, indicating XMU-MP-2 could dramatically enhance the sensitivity of chemotherapy in CRC.

## Conclusions

In conclusion, we established the essential role of the PTK6/JAK2/STAT3 axis in promoting the stemness and chemoresistance of CRC. The combined XMU-MP-2 treatment and chemotherapy with L-OHP had a potential in clinical CRC treatment.

## Supplementary Information

**Additional file 1: Supplementary Figure S1.** (A) Immunofluorescence staining was performed to verify the successful construction of PTK6 knockdown CRC cells. The scale bar represents 5 μm. (B) Representative flow cytometery images show the influence of PTK6 on the apoptosis of CRC cells. (C) Representative flow cytometery images demonstrate the effects of PTK6 on the cell cycle progression of CRC cells. (D) Cell viability analyses were performed in PTK6 silencing CRC cells after 5-FU and L-OHP treatment (mean ± SD, *n *= 3). **Supplementary Figure S2.** (A) The GSEA results indicate the enrichment of gene sets related to chemoresistance pathways in PTK6 overexpression CRC tissues from GSE73360. (B) The correlations of PTK6 with chemoresistance related genes were detected from TCGA database. (C) The in vitro limiting dilution assay shows the effects of PTK6 on the formation of CSC spheres. (mean ± SD, *n* = 12), likelihood ratio test. (D) Tumor sphere formation assays indicate that silencing of PTK6 decreases the sphere formation of CSC cells. Scale bar represents 100μm. The right panel shows the relative fold change of sphere number. (mean ± SD, *n *= 3) (E) Real-time q-PCR results demonstrate a reduced expression of chemoresistance related genes in PTK6 silencing CRC cells. (mean ± SD, *n *= 3) (F-G) Representative flow cytometery images show the distribution of the CD133+ and SOX2+ cells in control and PTK6 silencing CRC cells. (mean ± SD, *n* = 3). *P< 0.05, **P < 0.01, ***P < 0.001. **Supplementary Figure S3.** (A) Tumor sphere formation assays show the representative spheres formed by vector, WT, PTK6-KM and PTK6-YF overexpression CRC cells. Scale bar represents 50 μm. (B) Real-time qPCR results show the expression of stem cell markers in vector, WT, PTK6-KM and PTK6-YF overexpression CRC cells. (mean ± SD, *n* = 3) (C) Representative flow cytometery images show the distribution of the CD133+ and SOX2+ cells in vector, WT, PTK6-KM and PTK6-YF overexpression CRC cells. **Supplementary Figure S4.** (A) The correlations between the expression of STAT3 and stemness related genes were detected using the GEO database. (B) Kaplan-Meier curves of overall survival in CRC patients from Nanfang cohort were evaluated based on the expression of p-JAK2 and p-STAT3. The H-score was used as the cut-off to distinguish high and low protein expression. **Supplementary Figure S5.** (A) Western blot assays show the influence of XMU-MP-2 on the activation of JAK2/STAT3 pathway and the expression of stem cell markers. (B) The influence of XMU-MP-2 on the viability of CRC cells. (C) Clone formation assays show the influence of XMU-MP-2 on CRC cell proliferation in vector, WT, PTK6-KM and PTK6-YF overexpression CRC cells. (D) Representative flow cytometery images show the distribution of the CD133+ cells in vector, WT, PTK6-KM and PTK6-YF overexpression CRC cells. **Supplementary Figure S6.** (A) IHC staining of the subcutaneous tumors indicates that XMU-MP-2 can inhibit the PTK6 induced activation of JAK2/STAT3 signaling and the expression of stem cell markers. (B) Representative immunohistochemical (IHC) staining images of CRC tissues from the Nanfang cohort show a positive correlation between the expression of PTK6 and stem cell markers. (C) XMU-MP-2 suppresses the weight and volume of the subcutaneous tumors in the L-OHP treated tumor-bearing mice. Both the volume and weight of subcutaneous tumor were shown in the right panel (mean ± SD, *n* = 3). (D) IHC staining indicates that XMU-MP-2 can reduce the Ki67 index of control and L-OHP treated subcutaneous tumors.(E) XMU-MP-2 suppresses the weight and volume of the PDX tumors derived from CASE 2 in the L-OHP treated tumor-bearing mice. Both the volume and weight of subcutaneous tumor were shown in the right panel (mean ± SD, *n* = 3). (F) IHC staining indicates that XMU-MP-2 can reduce the phosphorylation of PTK6 and the stem cell markers in the PDX tumors derived from CASE2. **Supplementary Table S1. **RT-qPCR primer sequences for human genes.

## Data Availability

The datasets generated and/or analysed during the current study are not publicly available but are available from the corresponding author on reasonable request.

## Abbreviations

CRC: Colorectal carcinoma
PTK6: Protein tyrosine kinase 6
PDX: Patient-derived xenograft
FBS: Fetal bovine serum
STR: Short tandem repeat
PBS: Phosphate-buffered saline
WB: Western blot
TCGA: The Cancer Genome Atlas
IHC: Immunohistochemistry
IF: Immunofluorescence
NOD-SCID: Non-obeseDiabetes-scid
