# Supplementary material for: Pharmacological targeting PTK6 inhibits the JAK2/STAT3 sustained stemness and reverses chemoresistance of colorectal cancer
Source: J Exp Clin Cancer Res. 2021 Sep 22;40:297. doi: 10.1186/s13046-021-02059-6 (PMC8456648; doi:10.1186/s13046-021-02059-6)
Supplement: Supplementary file 1 — Additional file 1: Supplementary Figure S1. (A) Immunofluorescence staining was performed to verify the successful construction of PTK6 knockdown CRC cells. The scale bar represents 5 μm. (B) Representative flow cytometery images show the influence of PTK6 on the apoptosis of CRC cells. (C) Representative flow cytometery images demonstrate the effects of PTK6 on the cell cycle progression of CRC cells. (D) Cell viability analyses were performed in PTK6 silencing CRC cells after 5-FU and L-OHP treatment (mean ± SD, n = 3). Supplementary Figure S2. (A) The GSEA results indicate the enrichment of gene sets related to chemoresistance pathways in PTK6 overexpression CRC tissues from GSE73360. (B) The correlations of PTK6 with chemoresistance related genes were detected from TCGA database. (C) The in vitro limiting dilution assay shows the effects of PTK6 on the formation of CSC spheres. (mean ± SD, n = 12), likelihood ratio test. (D) Tumor sphere formation assays indicate that silencing of PTK6 decreases the sphere formation of CSC cells. Scale bar represents 100μm. The right panel shows the relative fold change of sphere number. (mean ± SD, n = 3) (E) Real-time q-PCR results demonstrate a reduced expression of chemoresistance related genes in PTK6 silencing CRC cells. (mean ± SD, n = 3) (F-G) Representative flow cytometery images show the distribution of the CD133+ and SOX2+ cells in control and PTK6 silencing CRC cells. (mean ± SD, n = 3). *P< 0.05, **P < 0.01, ***P < 0.001. Supplementary Figure S3. (A) Tumor sphere formation assays show the representative spheres formed by vector, WT, PTK6-KM and PTK6-YF overexpression CRC cells. Scale bar represents 50 μm. (B) Real-time qPCR results show the expression of stem cell markers in vector, WT, PTK6-KM and PTK6-YF overexpression CRC cells. (mean ± SD, n = 3) (C) Representative flow cytometery images show the distribution of the CD133+ and SOX2+ cells in vector, WT, PTK6-KM and PTK6-YF overexpression CRC cells. Supplementary [file 13046_2021_2059_MOESM1_ESM.docx]

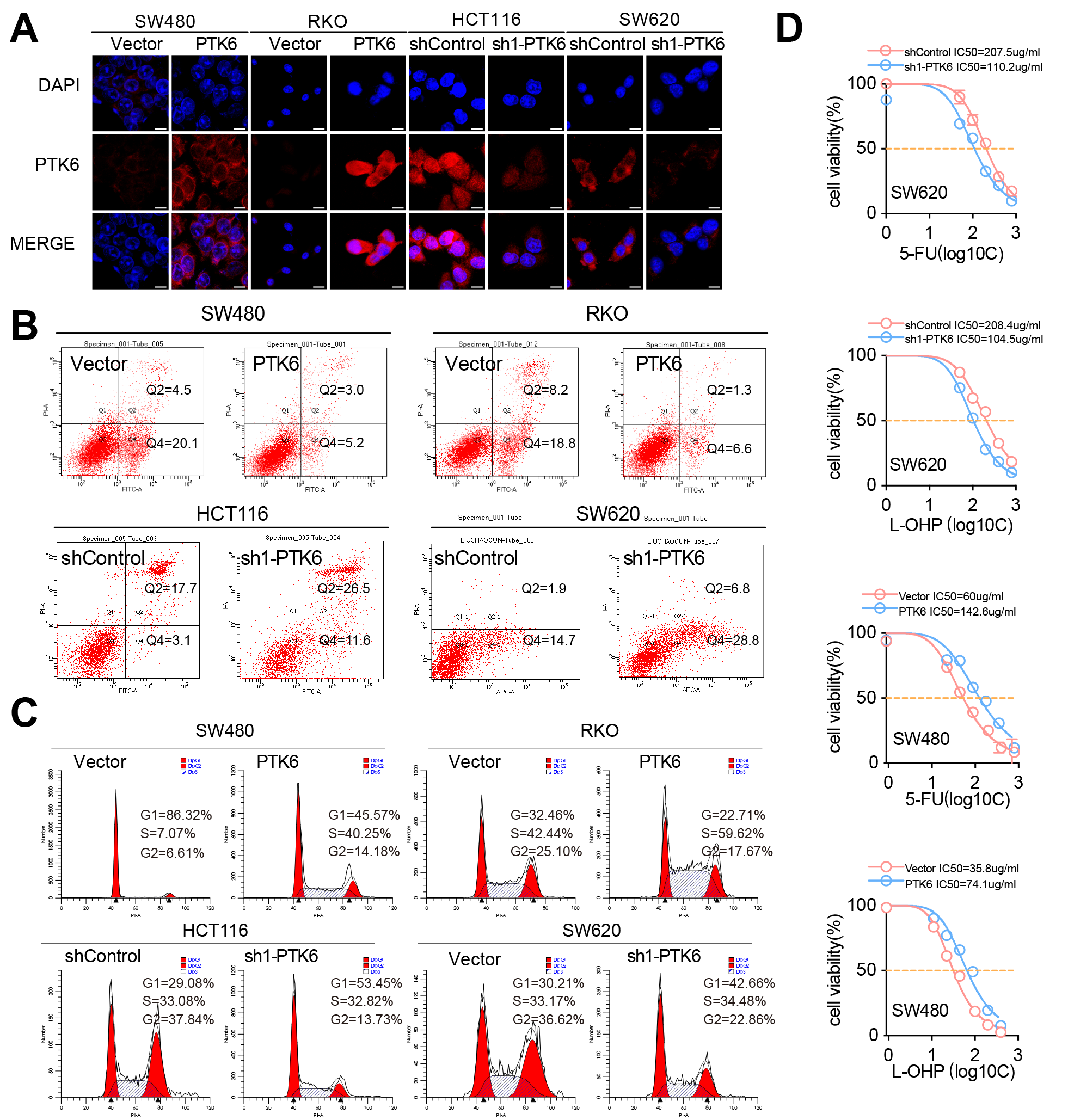


Supplementary Fig S1. (A) Immunofluorescence staining was performed to verify the successful construction of PTK6 knockdown CRC cells. The scale bar represents 5 μm. (B) Representative flow cytometery images show the influence of PTK6 on the apoptosis of CRC cells. (C) Representative flow cytometery images demonstrate the effects of PTK6 on the cell cycle progression of CRC cells. (D) Cell viability analyses were performed in PTK6 silencing CRC cells after 5-FU and L-OHP treatment(mean ± SD,n=3).


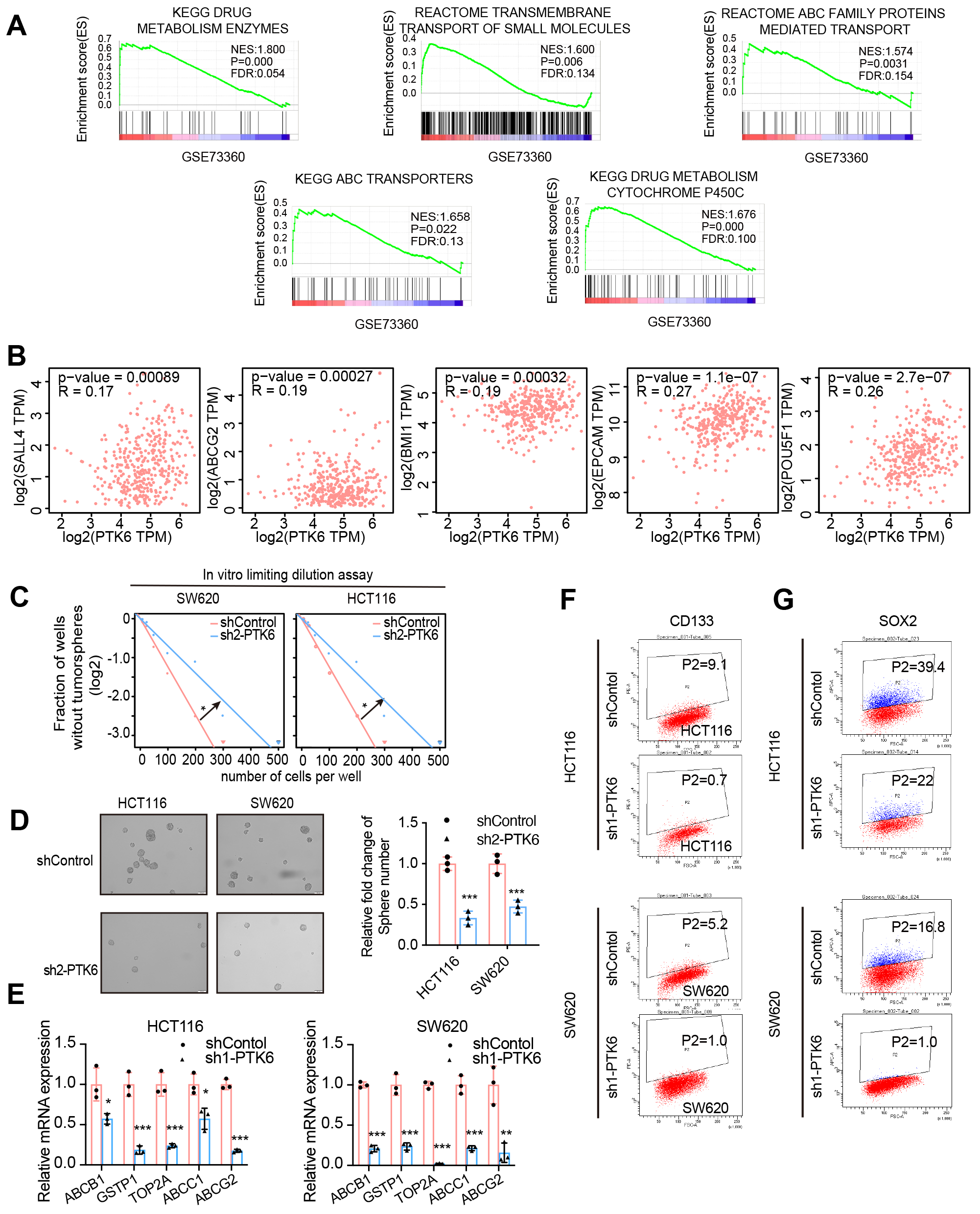


Supplementary Fig S2. (A) The GSEA results indicate the enrichment of gene sets related to chemoresistance pathways in PTK6 overexpression CRC tissues from GSE73360. (B) The correlations of PTK6 with chemoresistance related genes were detected from TCGA database. (C) The in vitro limiting dilution assay shows the effects of PTK6 on the formation of CSC spheres. (mean ± SD,n=12), likelihood ratio test. (D) Tumor sphere formation assays indicate that silencing of PTK6 decreases the sphere formation of CSC cells. Scale bar represents 100μm. The right panel shows the relative fold change of sphere number. (mean ± SD,n=3) (E) Real-time q-PCR results demonstrate a reduced expression of chemoresistance related genes in PTK6 silencing CRC cells. (mean ± SD,n=3) (F-G) Representative flow cytometery images show the distribution of the CD133+ and SOX2+ cells in control and PTK6 silencing CRC cells. (mean ± SD,n=3). *P < 0.05, **P < 0.01, ***P < 0.001


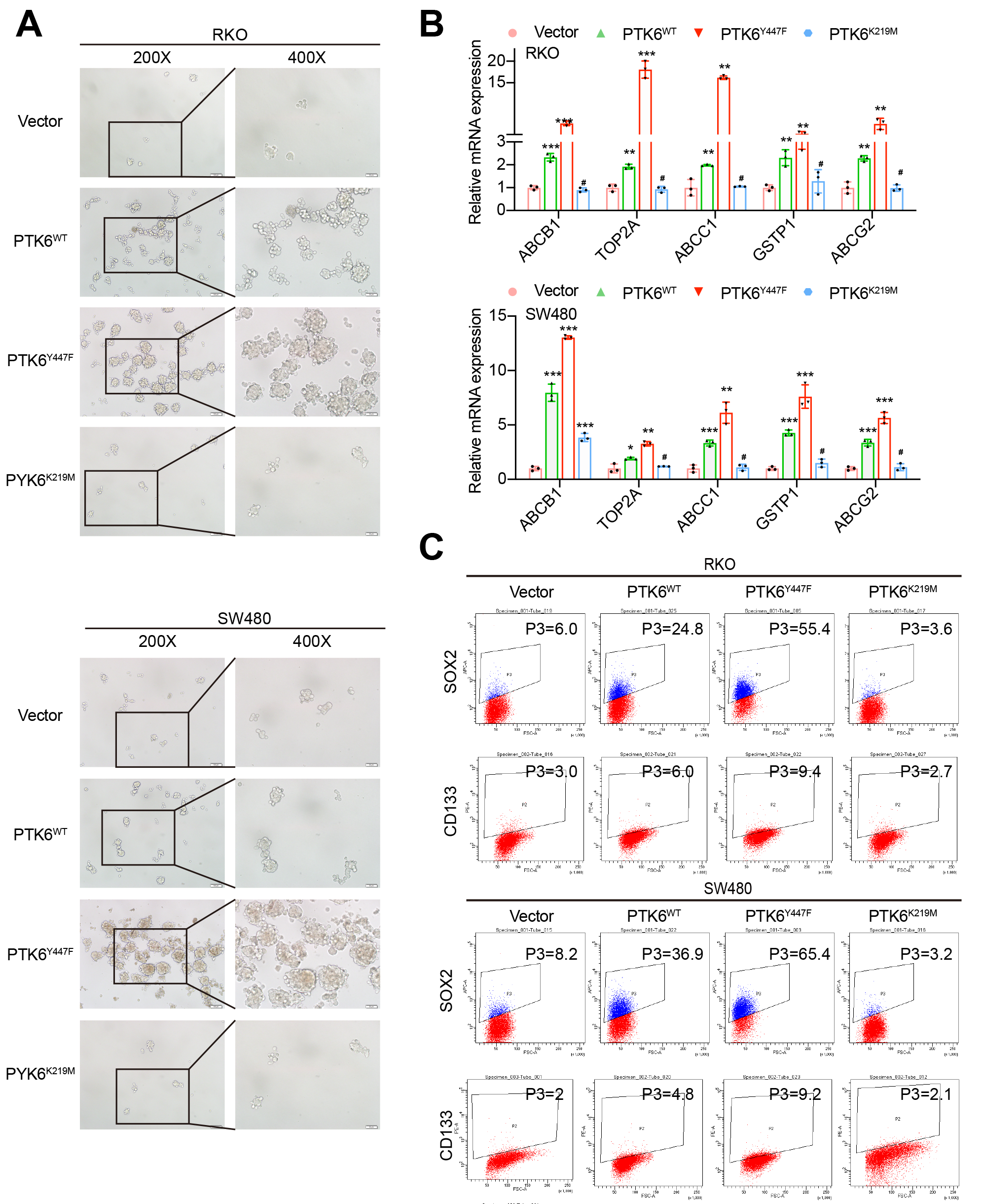


Supplementary Fig S3. (A) Tumor sphere formation assays show the representative spheres formed by vector, WT, PTK6-KM and PTK6-YF overexpression CRC cells.. Scale bar represents 50 μm. (B) Real-time qPCR results show the expression of stem cell markers in vector, WT, PTK6-KM and PTK6-YF overexpression CRC cells. (mean ± SD,n=3) (C) Representative flow cytometery images show the distribution of the CD133+ and SOX2+ cells in vector, WT, PTK6-KM and PTK6-YF overexpression CRC cells.


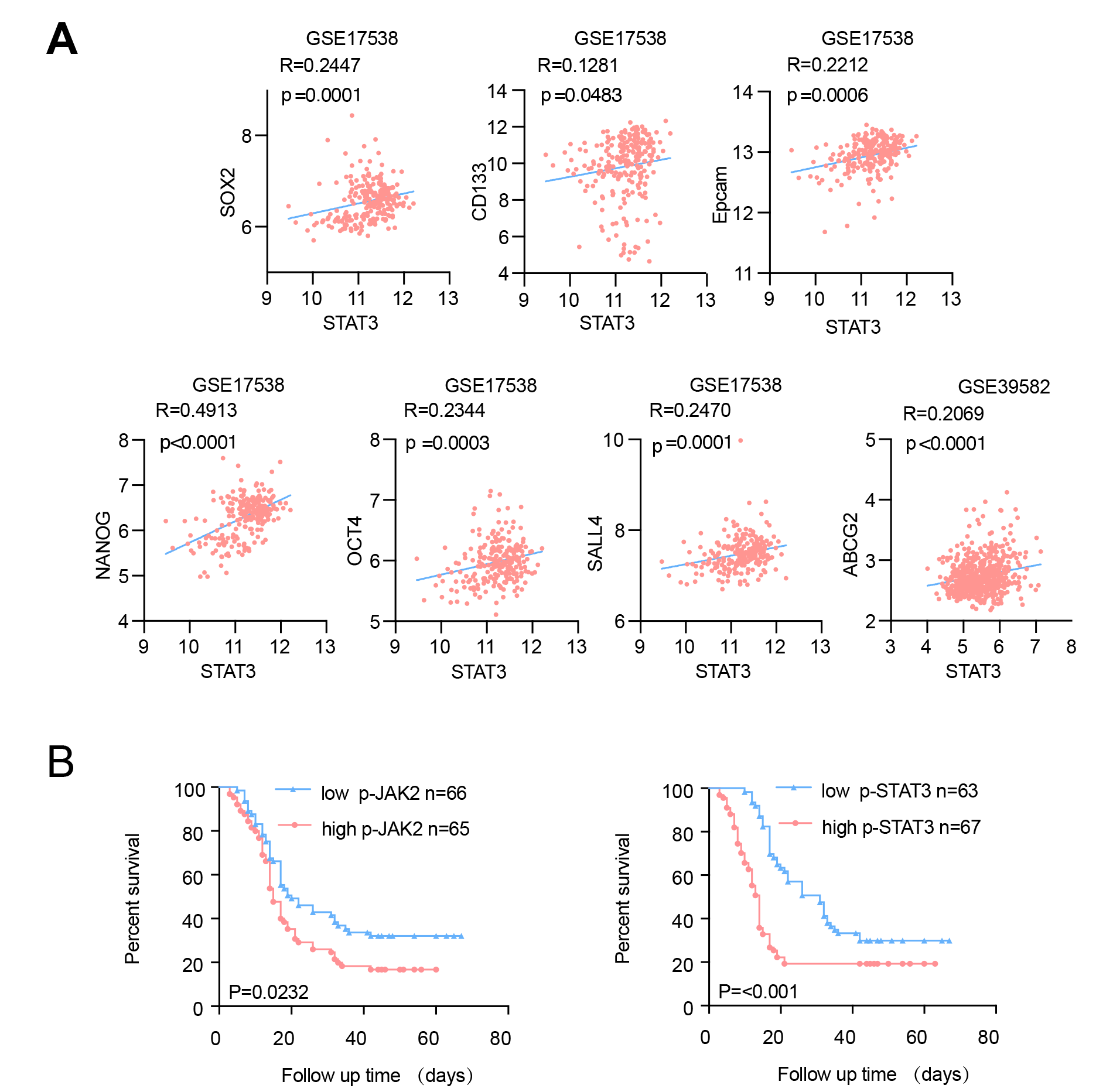


Supplementary Fig S4. (A) The correlations between the expression of STAT3 and stemness related genes were detected using the GEO database. (B) Kaplan-Meier curves of overall survival in CRC patients from Nanfang cohort were evaluated based on the expression of p-JAK2 and p-STAT3. The H-score was used as the cut-off to distinguish high and low protein expression.


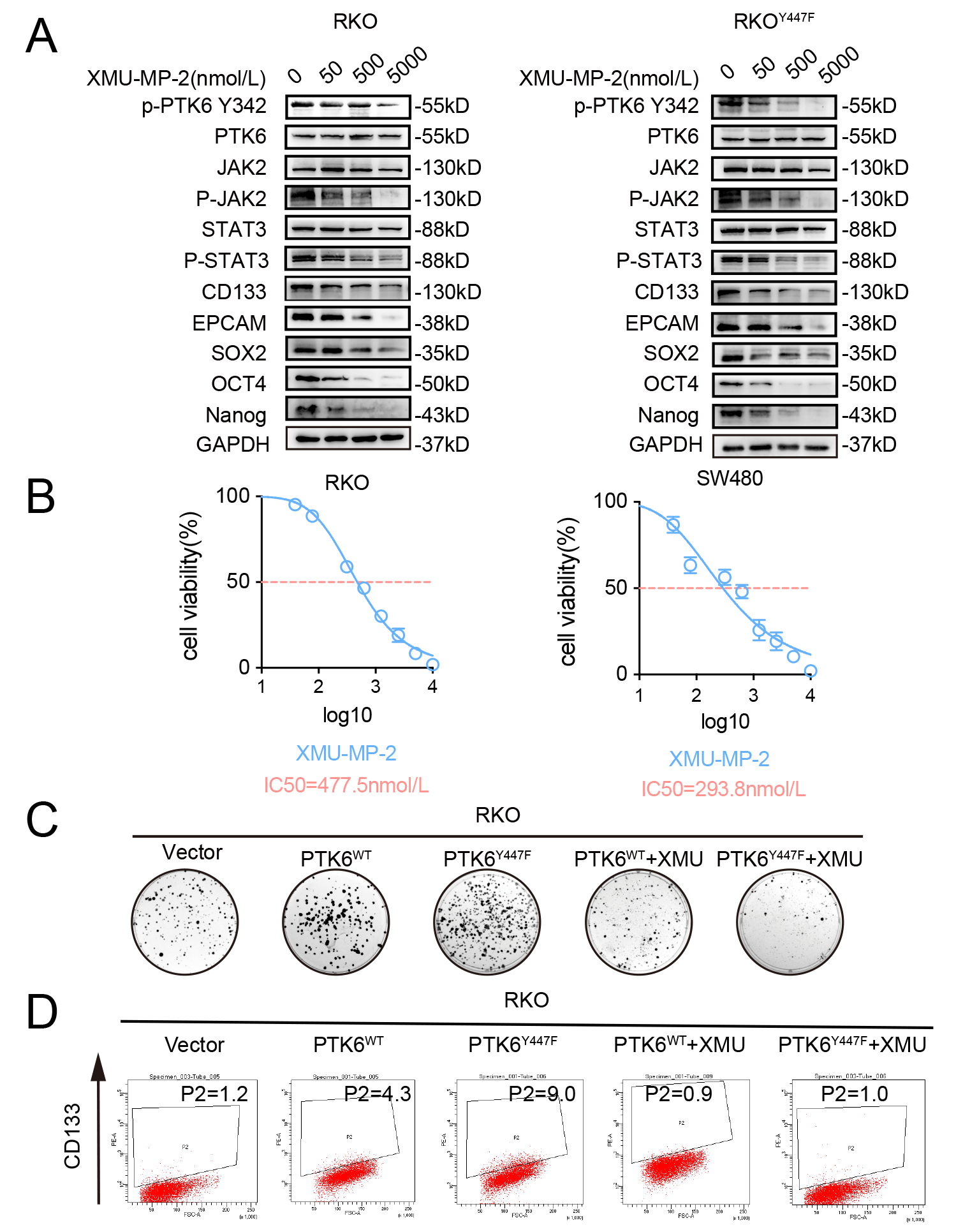


Supplementary Fig S5. (A) Western blot assays show the influence of XMU-MP-2 on the activation of JAK2/STAT3 pathway and the expression of stem cell markers. (B) The influence of XMU-MP-2 on the viability of CRC cells. (C) Clone formation assays show the influence of XMU-MP-2 on CRC cell proliferation in vector, WT, PTK6-KM and PTK6-YF overexpression CRC cells. (D) Representative flow cytometery images show the distribution of the CD133+ cells in vector, WT, PTK6-KM and PTK6-YF overexpression CRC cells.


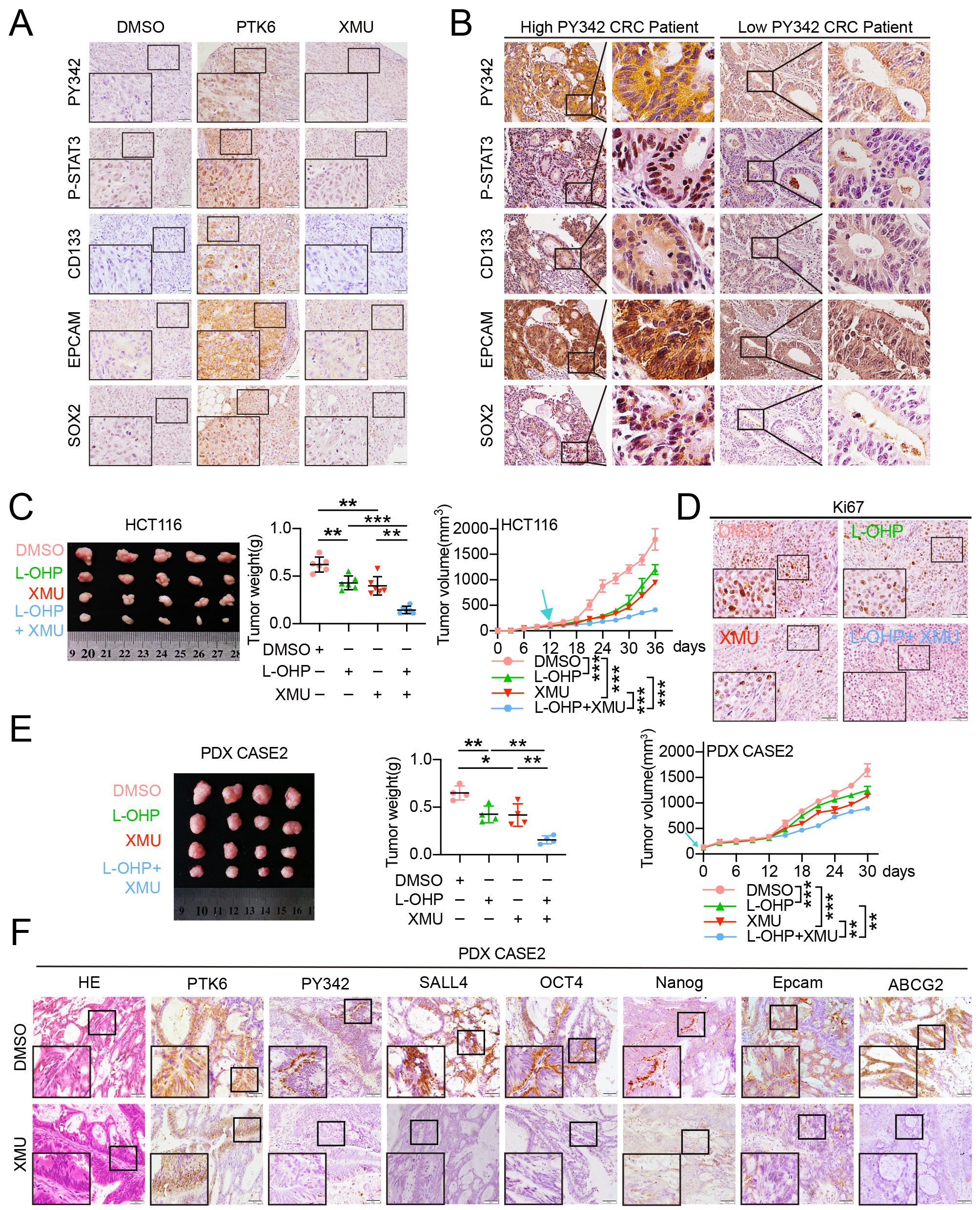


Supplementary Fig S6. (A) IHC staining of the subcutaneous tumors indicates that XMU-MP-2 can inhibit the PTK6 induced activation of JAK2/STAT3 signaling and the expression of stem cell markers. (B) Representative immunohistochemical (IHC) staining images of CRC tissues from the Nanfang cohort show a positive correlation between the expression of PTK6 and stem cell markers.(C) XMU-MP-2 suppresses the weight and volume of the subcutaneous tumors in the L-OHP treated tumor-bearing mice. Both the volume and weight of subcutaneous tumor were shown in the right panel . (mean ± SD,n=3) (D) IHC staining indicates that XMU-MP-2 can reduce the Ki67 index of control and L-OHP treated subcutaneous tumors.(E) XMU-MP-2 suppresses the weight and volume of the PDX tumors derived from CASE 2 in the L-OHP treated tumor-bearing mice. Both the volume and weight of subcutaneous tumor were shown in the right panel. (mean ± SD,n=3) (F) IHC staining indicates that XMU-MP-2 can reduce the phosphorylation of PTK6 and the stem cell markers in the PDX tumors derived from CASE2.

**Supplementary Table S1.** RT-qPCR primer sequences for human genes

| **Gene** | **Forward primer** | **Reverse primer** |
| --- | --- | --- |
| **PTK6** | 5'-CGGACCCGTGGTTCTTTG-3' | 5'-ACTCGGCTTCTCGCTGAC-3' |
| **GAPDH** | 5'-GGAGCGAGATCCCTCCAAAAT-3' | 5'-GGCTGTTGTCATACTTCTCATGG-3' |
| **CD133** | 5'-AGTCGGAAACTGGCAGATAGC-3' | 5'-GGTAGTGTTGTACTGGGCCAAT-3' |
| **EPCAM** | 5'-AATCGTCAATGCCAGTGTACTT-3' | 5'-TCTCATCGCAGTCAGGATCATAA-3' |
| **SALL4** | 5'-CCCGGCAGTAAGGACTGTC-3' | 5'-TCTCATCGCAGTCAGGATCATAA-3' |
| **BMI1** | 5'-GCTGCCAATGGCTCTAATGAA-3' | 5'-TGCTGGGCATCGTAAGTATCTT-3' |
| **SOX2** | 5'-TACAGCATGTCCTACTCGCAG-3' | 5'-GAGGAAGAGGTAACCACAGGG-3' |
| **NANOG** | 5'-CAGCCCTGATTCTTCTACCAG-3' | 5'-GATGCGTTCACCAGATAGCC-3' |
| **OCT4** | 5'-CAAAGCAGAAACCCTCGTGC-3' | 5'-TCTCACTCGGTTCTCGATACTG-3' |
| **ABCB1** | 5'-TTGCTGCTTACATTCAGGTTTCA-3' | 5'-AGCCTATCTCCTGTCGCATTA-3' |
| **GSTP1** | 5'-CCCTACACCGTGGTCTATTTCC-3' | 5'-CAGGAGGCTTTGAGTGAGC |
| **TOP2A** | 5'-ACCATTGCAGCCTGTAAATGA-3' | 5'-GGGCGGAGCAAAATATGTTCC-3' |
| **ABCC1** | 5'-CTCTATCTCTCCCGACATGACC-3' | 5'-AGCAGACGATCCACAGCAAAA-3' |
| **ABCG2** | 5'-ACGAACGGATTAACAGGGTCA-3' | 5'-CTCCAGACACACCACGGAT-3' |
